# Supplementary figures and images for: Nitroglycerin for treatment of retained placenta: A randomised, placebo-controlled, multicentre, double-blind trial in the UK
Source: PLoS Med. 2019 Dec 30;16(12):e1003001. doi: 10.1371/journal.pmed.1003001 (PMC6936786; doi:10.1371/journal.pmed.1003001)

S1 Figure


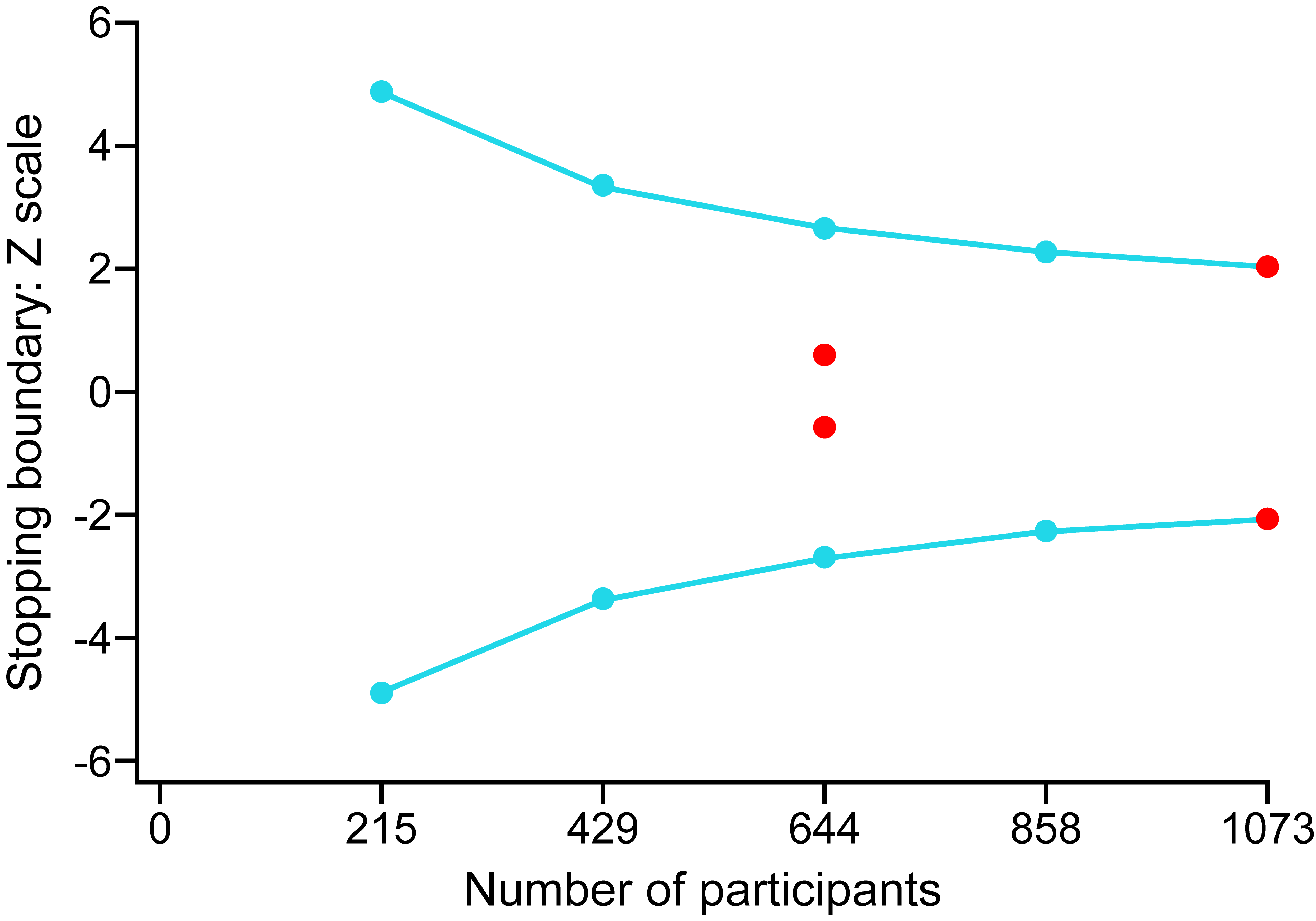
**Stopping boundaries**

Blue: efficacy boundaries; red: futility boundaries

Supplement: S1 Fig — (DOCX) [file pmed.1003001.s002.docx]
