# Supplementary material for: Nitroglycerin for treatment of retained placenta: A randomised, placebo-controlled, multicentre, double-blind trial in the UK
Source: PLoS Med. 2019 Dec 30;16(12):e1003001. doi: 10.1371/journal.pmed.1003001 (PMC6936786; doi:10.1371/journal.pmed.1003001)
Supplement: S1 Table — (DOCX) [file pmed.1003001.s003.docx]

**S1 Table: Recruitment by centre**

| **Centre** | **Randomised N=1107** | **Nitroglycerin**  **N=543** | **Placebo N=564** |
| --- | --- | --- | --- |
| Royal Infirmary of Edinburgh, Edinburgh | 92 (8.3) | 45 (8.3) | 47 (8.3) |
| Queen Elizabeth University Hospital, Glasgow | 24 (2.2) | 11 (2.0) | 13 (2.3) |
| St Mary's Hospital, Manchester | 122 (11.0) | 64 (11.8) | 58 (10.3) |
| Royal Victoria Infirmary, Newcastle | 42 (3.8) | 20 (3.7) | 22 (3.9) |
| Royal Preston Hospital, Preston | 80 (7.2) | 39 (7.2) | 41 (7.3) |
| Warrington Hospital, Warrington | 45 (4.1) | 21 (3.9) | 24 (4.3) |
| Chesterfield Royal Hospital, Chesterfield | 47 (4.2) | 21 (3.9) | 26 (4.6) |
| Leighton Hospital, Crewe | 46 (4.2) | 20 (3.7) | 26 (4.6) |
| University Hospital of North Durham, Durham | 33 (3.0) | 16 (2.9) | 17 (3.0) |
| West Middlesex University Hospital, West Middlesex | 28 (2.5) | 14 (2.6) | 14 (2.5) |
| Stoke Mandeville, Aylesbury | 55 (5.0) | 27 (5.0) | 28 (5.0) |
| Furness General Hospital, Furness | 10 (0.9) | 6 (1.1) | 4 (0.7) |
| University Hospital Southampton NHS Foundation Trust, Southampton | 47 (4.2) | 24 (4.4) | 23 (4.1) |
| Bolton NHS Foundation Trust, Bolton | 48 (4.3) | 24 (4.4) | 24 (4.3) |
| Sunderland Royal Hospital, Sunderland | 86 (7.8) | 42 (7.7) | 44 (7.8) |
| Oxford University Hospitals, Oxford | 45 (4.1) | 23 (4.2) | 22 (3.9) |
| Nottingham University Hospitals NHS Trust Queen's Medical Centre | 28 (2.5) | 13 (2.4) | 15 (2.7) |
| Nottingham University Hospitals NHS Trust City Campus | 37 (3.3) | 18 (3.3) | 19 (3.4) |
| East Lancashire Hospital NHS Trust Burnley General Hospital, Burnley | 28 (2.5) | 13 (2.4) | 15 (2.7) |
| Ashford and St Peter's Hospitals NHS Trust, Chertsey | 17 (1.5) | 9 (1.7) | 8 (1.4) |
| North Tees and Hartlepool Hospitals NHS Foundation Trust, Stockton-on-Tees | 19 (1.7) | 10 (1.8) | 9 (1.6) |
| South Tees Hospital NHS Foundation Trust James Cook Hospital, Middlesborough | 17 (1.5) | 9 (1.7) | 8 (1.4) |
| Countess of Chester Hospital NHS Foundation Trust, Chester | 18 (1.6) | 8 (1.5) | 10 (1.8) |
| Darlington Memorial Hospital, Darlington | 8 (0.7) | 4 (0.7) | 4 (0.7) |
| York Teaching Hospital, York | 21 (1.9) | 9 (1.7) | 12 (2.1) |
| The Royal Berkshire Hospital, Reading | 16 (1.4) | 8 (1.5) | 8 (1.4) |
| Milton Keynes University Hospital, Milton Keynes | 20 (1.8) | 12 (2.2) | 8 (1.4) |
| Shrewsbury and Telford Hospital NHS Trust, Telford | 18 (1.6) | 8 (1.5) | 10 (1.8) |
| Frimley Park, Frimley | 10 (0.9) | 5 (0.9) | 5 (0.9) |

Values are n (%)
