# Supplementary material for: Nitroglycerin for treatment of retained placenta: A randomised, placebo-controlled, multicentre, double-blind trial in the UK
Source: PLoS Med. 2019 Dec 30;16(12):e1003001. doi: 10.1371/journal.pmed.1003001 (PMC6936786; doi:10.1371/journal.pmed.1003001)
Supplement: S2 Table — (DOCX) [file pmed.1003001.s004.docx]

**S2_Table:**

**Identification, measurement and valuation of resource use**

| **Resource** | **How Measured?** | **Unit cost** | **Source of Valuation** |
| --- | --- | --- | --- |
| **Hospital Episode Resources** |  |  |  |
| Nitroglycerin | Drug (2 x 400mcg/ puff) administered or not | £3.44/ 200-dose unit | British National Formulary^1^ |
| Monitoring of Patient by Hospital | Length of time to placenta delivery/ or time transfer to theatre | £108/ hr of patient contact | Unit costs of health and social care 2016^2^ |
| Management by surgical team in theatre (for those with spontaneous delivery of placenta) | Length of time in theatre | £667 / hr | Information Services Division Scotland^3^ |
| Hospital stay (following spontaneous delivery of placenta) | Length of stay (no. of days) | £454.86/ day | Healthcare resource group codes (*NES/ XS for †NZ27Z)^4^ |
| Manual removal of placenta | Manual removal of placenta performed or not | Daycase £983.38;  Non-elective short-stay (1 day)) £1,149.59;  Non-elective (> 1 day) £1, 149.59 + (£496.61 x No. of days >1 day) | Health resource group codes (†NZ27, ‡OPCS:  R29.1)^4^ |
| Blood Transfusion | Blood transfusion administered or not | £332.15/ 2 red cell unit per patient | National costing statement on blood transfusion 2015^5^ |
| **Primary Care Resources** |  |  |  |
| Midwife calls | Number of calls made to midwife | £7.33/10-min per call | Unit costs of health and social care 2016^2^ |
| Midwife visits | Number of midwife visits | £14.67/20-min per visit | Unit costs of health and social care 2016^2^ |
| Health visitor calls | Number of calls made to health visitor | £7.00/ 10-min per call | Unit costs of health and social care 2016^2^ |
| Health visitor visits | Number of health visitor visits | £14/ 20-min per visit | Unit costs of health and social care 2016^2^ |
| General Practitioner visits | Number of visits to General Practitioner | £36/9.22-min per visit | Unit costs of health and social care 2016^2^ |
| General Practitioner calls | Number of telephone consultations with General Practitioner | £36/9.22-min per call | Unit costs of health and social care 2016^2^ |
| General Practitioner home visits | Number of home visits by General Practitioner | £44.84/ 11.4-min per home visit | Unit costs of health and social care 2016^2^ |
| **Secondary Care Resources** |  |  |  |
| Outpatient Attendance | Number of outpatient appointments | £136.79 per attendance | Unit costs of health and social care 2016^2^ |
| Hospital re-admission | Length of stay (no. of days) | Various | ^τ^HRG based reference costs^4^ |

Notes: The costing is made on the basis of comparing nitroglycerin to no treatment;

*NES/XS – Non-elective short stay, excess bed-days

†NZ27Z – Post-natal therapeutic procedures

‡OPCS R29.1 - The OPCS Classification of Interventions and Procedures version 4 for retained placenta.

^τ^HRG- Healthcare Resource Group
