# Supplementary material for: Nitroglycerin for treatment of retained placenta: A randomised, placebo-controlled, multicentre, double-blind trial in the UK
Source: PLoS Med. 2019 Dec 30;16(12):e1003001. doi: 10.1371/journal.pmed.1003001 (PMC6936786; doi:10.1371/journal.pmed.1003001)
Supplement: S3 Table — (DOCX) [file pmed.1003001.s005.docx]

**S3_Table**

**Description of participants who were excluded pre-randomisation**

|  | n | % |
| --- | --- | --- |
| **Patients screened not eligible for trial** | **483** |  |
| Reasons for ineligibility | **353** |  |
| Did not meet eligibility criteria | 335 | 94.9 |
| Clinical reasons | 12 | 3.4 |
| Placenta delivered before eligible criteria could be checked | 6 | 1.1 |
| Reasons for declining to take part | **63** |  |
| No reason given | 22 | 34.9 |
| Prefers to go straight to theatre | 15 | 23.8 |
| Does not wish to take part | 11 | 17.5 |
| Did not want to participate in research | 5 | 7.9 |
| Randomisation process | 2 | 3.2 |
| Other | 8 | 12.7 |
| Reasons for missing participants | **60** |  |
| No medical staff on duty that were on the delegation log | 1 | 1.6 |
| No reason given | 40 | 66.7 |
| Recruitment on halt | 15 | 25.0 |
| Drugs not available | 4 | 6.7 |
| Not appropriate to approach due to clinical reasons | **7** |  |
