# Supplementary material for: Nitroglycerin for treatment of retained placenta: A randomised, placebo-controlled, multicentre, double-blind trial in the UK
Source: PLoS Med. 2019 Dec 30;16(12):e1003001. doi: 10.1371/journal.pmed.1003001 (PMC6936786; doi:10.1371/journal.pmed.1003001)
Supplement: S4 Table — (DOCX) [file pmed.1003001.s006.docx]

**S4_Table**

**Reasons why study drug was not given**

| Reasons study drug not given | **Nitroglycerin N=542** | **Placebo**  **N=562** |
| --- | --- | --- |
|  | 6/542 (1.1) | 7/562 (1.2) |
| Placenta was delivered before drug was administered^1^ | 0 | 4 |
| Systolic blood pressure ≤100 mmHg^1^ | 2 | 1 |
| Systolic blood pressure ≤100 mmHg and pulse >119bpm^1^ | 2 | 0 |
| Pulse >119bpm^1^ | 0 | 1 |
| Bleeding^1^ | 1 | 1 |
| Medical team wrongly assuming spray had been given | 1 | 0 |

^1^Observations taken immediately before drug administration meant that the woman was no longer

eligible for trial inclusion. Drug was not given on safety grounds.
