# Supplementary material for: Nitroglycerin for treatment of retained placenta: A randomised, placebo-controlled, multicentre, double-blind trial in the UK
Source: PLoS Med. 2019 Dec 30;16(12):e1003001. doi: 10.1371/journal.pmed.1003001 (PMC6936786; doi:10.1371/journal.pmed.1003001)
Supplement: S5 Table — (DOCX) [file pmed.1003001.s007.docx]

**S5_Table**

**Method of placenta removal**

|  | **Nitroglycerin**  **N=541** | **Placebo**  **N=563** |
| --- | --- | --- |
| **Placenta delivered within 15 minutes** |  |  |
| Spontaneous | 5/36 (13.9) | 6/45 (13.3) |
| Controlled cord traction | 31/36 (86.1) | 39/45 (86.7) |
| **Placenta delivered after 15 minutes** |  |  |
| Spontaneous | 13/505 (2.6) | 16/518 (3.1) |
| Controlled cord traction | 83/505 (16.4) | 84/518 (16.2) |
| Manual removal of placenta | 407/505 (80.6) | 417/518 (80.5) |
| Other^1^ | 1/505 (0.2) | 1/518 (0.2) |
| Unknown^2^ | 1/505 (0.2) |  |

^1^Suction removal; removed with uterus at hysterectomy. ^2^Participant was discharged with placenta still inside
