# Supplementary material for: Nitroglycerin for treatment of retained placenta: A randomised, placebo-controlled, multicentre, double-blind trial in the UK
Source: PLoS Med. 2019 Dec 30;16(12):e1003001. doi: 10.1371/journal.pmed.1003001 (PMC6936786; doi:10.1371/journal.pmed.1003001)
Supplement: S6 Table — (DOCX) [file pmed.1003001.s008.docx]

**S6_Table**

**Resource use by treatment allocation (intention to treat)**

| **Resource Use** | **No. of Observations** | **N=1104*** | |
| --- | --- | --- | --- |
|  |  | **Nitroglycerin**  **(n=541)** | **Placebo**  **(n=563)** |
| **Type of Hospital Resources (Initial Episode)** |  |  |  |
| Time to Placenta Delivery, Hours; mean (SD) | 1087 | 1.31 (0.81) | 1.28 (0.77) |
| Time to Theatre, Hours; Mean (SD) | 1065 | 0.79 (0.73) | 0.75 (0.66) |
| Time in Theatre, Hours; Mean (SD) | 1000 | 0.82 (0.75) | 0.82 (0.71) |
| Method of Placenta Removal | 1104 |  |  |
| Non-manual removal of placenta (spontaneous, controlled cord traction); n (%) |  | 134 (24.8%) | 146 (25.9%) |
| Manual removal of placenta; n (%) |  | 407 (75.2%) | 417 (74.1%) |
| Blood transfusion; n (%) | 1084 | 61 (11.4) | 43 (7.8) |
| Length of Stay, Hours; Mean (SD) | 1014 | 45.46 (35.33) | 42.74 (29.81) |
| **Type of Primary Care Resources** |  |  |  |
| Number of calls to midwife | 460 | 0.30 (1.12) | 0.17 (0.46) |
| Number of midwife visits | 457 | 0.24 (0.90) | 0.28 (0.93) |
| Number of calls made to health visitor | 452 | 0.04 (0.37) | 0.07 (0.39) |
| Number of health visitor visits | 452 | 0.10 (0.52) | 0.18 (0.80) |
| Number of visits to general practitioner | 457 | 0.38 (0.68) | 0.49 (0.96) |
| Number of general practitioner telephone consultation | 446 | 0.18 (0.50) | 0.17 (0.51) |
| Number of home visits by general practitioner | 436 | 0.03 (0.19) | 0.004 (0.07) |
| **Type of Secondary Care Resources** |  |  |  |
| No. of Outpatient Attendance; Mean (SD) | 466 | 0.19 (0.72) | 0.14 (0.47) |
| Readmission | 1098 |  |  |
| Yes; n (%) |  | 16 (3.0%) | 29 (5.2%) |
| No; n (%) |  | 520 (97%) | 533 (94.8%) |
| Average length of stay if readmitted (No. of Nights); Mean (SD) | 45 | 3.94 (13.12) | 1.31 (1.85) |

* 13 individuals were not given drug (7 in the placebo group and 6 in the GTN group)
