# Supplementary material for: Nitroglycerin for treatment of retained placenta: A randomised, placebo-controlled, multicentre, double-blind trial in the UK
Source: PLoS Med. 2019 Dec 30;16(12):e1003001. doi: 10.1371/journal.pmed.1003001 (PMC6936786; doi:10.1371/journal.pmed.1003001)
Supplement: S7 Table — (DOCX) [file pmed.1003001.s009.docx]

**S7 Table**

**Severe Adverse Events**

|  | **Nitroglycerin, N=541** | **Placebo, N=563** |
| --- | --- | --- |
| Number of Severe Adverse Events* | 27 (5.0) | 25 (4.4) |
| Hospitalisation | 24 | 24 |
| Life-threatening | 0 | 3 |
| Other significant medical event | 6 | 2 |
| Severity of event |  |  |
| Mild | 7 | 4 |
| Moderate | 19 | 16 |
| Severe | 1 | 5 |
| Event due to progression of underlying disease |  |  |
| Yes | 5 | 2 |
| No | 21 | 23 |
| Not applicable | 1 | 0 |
| Event due to a lack of efficacy of investigational medicinal product | |  |
| Yes | 1 | 0 |
| No | 25 | 25 |
| Not applicable | 1 | 0 |
| Details of Severe Adverse Event |  |  |
| Postpartum haemorrhage | 23 | 16 |
| Retained products of conception | 1 | 3 |
| Endometritis | 1 | 0 |
| Sepsis | 0 | 1 |
| Chest infection | 1 | 0 |
| Gall stone pancreatitis | 1 | 0 |
| Anaphylaxis due to suxamethonium | 0 | 1 |
| Escherichia coli and clostridium difficile infection | 0 | 1 |
| Elevated blood pressure | 0 | 1 |
| Emergency hysterectomy | 0 | 1 |
| Post Dural headache | 0 | 1 |

*Participants only experienced one Serious Adverse Event. Values are n (%) or n.
