# Supplementary material for: Nitroglycerin for treatment of retained placenta: A randomised, placebo-controlled, multicentre, double-blind trial in the UK
Source: PLoS Med. 2019 Dec 30;16(12):e1003001. doi: 10.1371/journal.pmed.1003001 (PMC6936786; doi:10.1371/journal.pmed.1003001)
Supplement: S1 Text — (PDF) [file pmed.1003001.s010.pdf]

≤

Study Protocol

A pragmatic group sequential placebo controlled randomised trial to determine the effectiveness of Glyceryl trinitrate for retained placenta.

Text

GOT-IT TRIAL

Glyceryl trinitrate fOr reTaIned placenTa

Co-sponsors	University of Edinburgh & NHS Lothian<br>ACCORD<br>The Queen's Medical Research Institute<br>47 Little France Crescent<br>Edinburgh<br>EH16 4TJ
Funder	NIHR HTA reference 12/29/01
Funding Reference Number	12/29/01
Chief Investigator	Dr Fiona Denison
EudraCT Number	2013-003810-42
REC Number	13/NE/0339
ISRCTN Number	ISRCTN88609453
Version Number and Date	Version 8 - 27 April 2016

COORDINATING CENTRE

**Chief Investigator**<br><br>Dr Fiona Denison<br><br>MRC Centre for Reproductive Health (Room W1.04)<br>Queen's Medical Research Institute<br>47 Little France Crescent<br>Edinburgh EH16 4TJ<br><br>Tel: 0131 242 6449<br>Fax: 0131 242 6441<br>Email: fiona.denison@ed.ac.uk	**Co-Sponsor Representative**<br><br>Dr Fiach O'Mahony<br><br>University of Edinburgh<br>ACCORD<br>The Queen's Medical Research Institute<br>47 Little France Crescent<br>Edinburgh EH16 4TJ<br><br>Tel: 0131 242 9418<br>Fax: 0131 242 9447<br>Email: Fiach.O'Mahony@ed.ac.uk
**Trial Manager**<br><br>Kathryn Carruthers<br>Room W1.14<br>The Queen's Medical Research Institute<br>47 Little France Crescent<br>Edinburgh EH16 4TJ<br><br>Tel: 0131 242 6753<br>Fax: 0131 242 6441<br><br>Email: Kathryn.Carruthers@ed.ac.uk	**Trial Administrator**<br><br>Jayne Richards<br>Room W1.14<br>The Queen's Medical Research Institute<br>47 Little France Crescent<br>Edinburgh EH16 4TJ<br><br>Tel: 0131 242<br>Fax: 0131 242 6441<br><br>Email: got-it@ed.ac.uk
**Co-Investigator**<br><br>Prof Jane Norman<br><br>Tommy's Centre for Maternal and Fetal Health<br>University of Edinburgh MRC Centre for Reproductive Health<br>Queen's Medical Research Centre<br>47 Little France Crescent<br>Edinburgh EH16 4TY<br><br>Tel: 0131 242 2694<br>Fax: 0131 242 6441<br>Email: jane.norman@ed.ac.uk	**Trial Statistician**<br><br>Professor John Norrie (PA: Lynn McKenzie)<br>Director, Centre for Healthcare Randomised Trials (CHaRT)<br>Health Services Research Unit<br>3rd Floor Health Sciences Building<br>University of Aberdeen<br>Foresterhill<br>Aberdeen AB25 2ZD<br><br>Tel: 01224 438 149 / 01224 438 198<br>Email: j.norrie@abdn.ac.uk / lynn.mckenzie@abdn.ac.uk
**Co-Investigator**<br><br>Dr Graham Scotland<br><br>Health Economics Research Unit<br>Institute of Applied Health Sciences<br>University of Aberdeen<br>Foresterhill<br>Aberdeen AB25 2ZD<br><br>Tel: 01224 437170<br>Fax:<br>Email: g.scotland @abdn.ac.uk	**Co-Investigator**<br><br>Prof Julia Lawton<br><br>Professor of Health and Social Science<br>Centre for Population Health Sciences<br>The University of Edinburgh<br>Medical School<br>Teviot Place<br>Edinburgh EH8 9AG<br><br>Tel: 0131 650 6197<br><br>Fax: 0131 650 6902<br>Email: j.lawton@ed.ac.uk
**Co-Investigator**	**Co-Investigator**

Ms Sheonagh Brook-Smith<br><br>Charge Midwife<br>Lothian Birth Centre<br>Edinburgh Royal Infirmary<br>51 Little France Crescent<br>Edinburgh EH16 4SA<br><br>Tel: 0131 242 2675 or 0131 242 2671<br>Email: sheonagh.brook-smith@nhslothian.scot.nhs.uk	Dr Gladys McPherson<br><br>Centre for Healthcare Randomised Trials (CHaRT)<br>Health Services Research Unit<br>3rd Floor Health Sciences Building<br>University of Aberdeen<br>Foresterhill<br>Aberdeen AB25 2ZD<br><br>Tel: 01224 438176<br>Fax: 01224 554580<br>Email: g.mcpherson@abdn.ac.uk
**Lay Applicant**<br><br>Ms Cynthia Clarkson	**Lay Applicant**<br><br>Mrs Mathilde Peace
**Lay Applicant**<br><br>Mrs Jane Brewin	

CONTENTS

1 **INTRODUCTION** 10
1.1 BACKGROUND 10
1.2 RATIONALE FOR STUDY 10
1.2.1 Rationale for GTN (nitric oxide donor) for RP 10
1.2.2 Biological plausibility of GTN for management of RP 10
1.2.3 Summary 11
2 **STUDY OBJECTIVES** 11
2.1 OBJECTIVES 11
2.1.1 Primary Objective 11
2.1.2 Secondary Objectives 11
2.2 OUTCOMES 12
2.2.1 Primary Outcomes 12
2.2.2 Secondary Outcomes 12
3 **STUDY DESIGN** 13
3.1 OVERVIEW 13
3.2 INTERNAL PILOT RCT 13
3.2.1 Detailed Design of Internal Pilot RCT 13
3.3 NESTED QUALITATIVE STUDY 13
3.3.1 Study Design 13
3.3.2 Aims 14
3.3.3 Objectives 14
3.3.4 Research Questions 14
3.3.5 Sample and Recruitment 14
3.3.6 Data Collection 14
3.3.7 Data Analysis 14
3.3.8 Implementation of Recommendations from Qualitative Research . 15
3.4 SUBSTANTIVE RCT 15
3.4.1 Detailed Design of Substantive RCT 15
3.4.2 Economic Evaluation 16
4 **STUDY POPULATION** 17
4.1 SETTING 17
4.2 NUMBER OF PARTICIPANTS 17
4.2.1 Internal Pilot RCT 17
4.2.2 Substantive RCT 17
4.2.3 Projected Trial Duration 17
4.3 INCLUSION CRITERIA 17
4.4 EXCLUSION CRITERIA 18
4.5 CO-ENROLMENT 18
5 **PARTICIPANT SELECTION AND ENROLMENT** 18
5.1 IDENTIFYING PARTICIPANTS 18
5.2 CONSENTING PARTICIPANTS 19
5.3 SCREENING FOR ELIGIBILITY 19
5.4 INELIGIBLE AND NON-RECRUITED PARTICIPANTS 19
5.5 RANDOMISATION 19
5.5.1 Randomisation Procedures 19

5.5.2 Treatment Allocation 19
5.5.3 Emergency Unblinding Procedures 19
5.5.4 Withdrawal of Study Participants 20

6 INVESTIGATIONAL MEDICINAL PRODUCT AND PLACEBO 20

6.1 STUDY DRUG 20
6.1.1 Study Drug Identification 20
6.1.2 Study Drug Manufacturer 20
6.1.3 Marketing Authorisation Holder 20
6.1.4 Labelling and Packaging 21
6.1.5 Storage 21
6.1.6 Summary of Product Characteristics 21
6.2 PLACEBO 21
6.3 DOSING REGIME 21
6.4 DOSE CHANGES 21
6.5 PARTICIPANT COMPLIANCE 21
6.6 OVERDOSE 21
6.7 OTHER MEDICATIONS 21
6.7.1 Non-Investigational Medicinal Products 21
6.7.2 Permitted Medications 22
6.7.3 Prohibited Medications 23

7 STUDY ASSESSMENTS 23

7.1 SAFETY ASSESSMENTS 23
7.2 STUDY ASSESSMENTS 23

8 DATA COLLECTION 24

9 STATISTICS AND DATA ANALYSIS 24

9.1 SAMPLE SIZE CALCULATION 24
9.2 PROPOSED ANALYSES 25

10 ADVERSE EVENTS 25

10.1 DEFINITIONS 25
10.2 IDENTIFYING AEs AND SAEs 26
10.3 RECORDING AEs AND SAEs 26
10.4 ASSESSMENT OF AEs AND SAEs 27
10.4.1 Assessment of Seriousness 27
10.4.2 Assessment of Causality 27
10.4.3 Assessment of Expectedness 27
10.4.4 Assessment of Severity 27
10.5 REPORTING OF SAEs/SARs/SUSARs 28
10.6 REGULATORY REPORTING REQUIREMENTS 28
10.7 FOLLOW UP PROCEDURES 28

11 PREGNANCY 28

12 TRIAL MANAGEMENT AND OVERSIGHT ARRANGEMENTS 29

12.1 PROJECT MANAGEMENT GROUP 29
12.2 TRIAL STEERING COMMITTEE 29
12.3 DATA MONITORING COMMITTEE 29
12.4 INSPECTION OF RECORDS 29
12.5 RISK ASSESSMENT 29
12.6 STUDY MONITORING AND AUDIT 29

13 GOOD CLINICAL PRACTICE ... 30
13.1 ETHICAL CONDUCT ... 30
13.2 REGULATORY COMPLIANCE ... 30
13.3 INVESTIGATOR RESPONSIBILITIES ... 30
13.3.1 Informed Consent ... 30
13.3.2 Study Site Staff ... 30
13.3.3 Data Recording ... 30
13.3.4 Investigator Documentation ... 31
13.3.5 GCP Training ... 31
13.3.6 Confidentiality ... 31
13.3.7 Data Protection ... 31
14 STUDY CONDUCT RESPONSIBILITIES ... 31
14.1 PROTOCOL AMENDMENTS ... 31
14.2 PROTOCOL VIOLATIONS AND DEVIATIONS ... 31
14.3 SERIOUS BREACH REQUIREMENTS ... 32
14.4 STUDY RECORD RETENTION ... 32
14.5 END OF STUDY ... 32
14.6 CONTINUATION OF DRUG FOLLOWING THE END OF STUDY ... 32
14.7 INSURANCE AND INDEMNITY ... 32
15 REPORTING, PUBLICATIONS AND NOTIFICATION OF RESULTS ... 33
15.1 AUTHORSHIP POLICY ... 33
15.2 PUBLICATION ... 33
15.3 PEER REVIEW ... 33
16 REFERENCES ... 34

PROTOCOL APPROVAL

GOT-IT TRIAL: A pragmatic group sequential placebo controlled randomised trial to determine the effectiveness of Glyceryl trinitrate for retained placenta.

EudraCT number: 2013-003810-42

Signatures:

Fiona Denison<br>Chief Investigator	Signature	27 April 2016<br>Date
John Norrie<br>Trial Statistician	Signature	Date
Fiach O'Mahony<br>Sponsor(s) Representative	Signature	Date
Name:__________________<br>Principal Investigator	Signature	Date

LIST OF ABBREVIATIONS

ACCORD	Academic and Clinical Central Office for Research & Development - Joint office for University of Edinburgh and NHS Lothian
AE	Adverse Event
AR	Adverse Reaction
BP	Blood Pressure
CRF	Case Report Form
CHaRT	Centre for Healthcare Randomised Trials
CI	Chief Investigator
CTA	Clinical Trials Authorisation
DMC	Data Monitoring Committee
GCP	Good Clinical Practice
GTN	Glyceryl Trinitrate
HTA	Health Technology Assessment
ICH	International Conference on Harmonisation
IMP	Investigational Medicinal Product
ISF	Investigator Site File
IVRS	Interactive Voice Response System
MROP	Manual removal of placenta
NHS	National Health Service
NICE	National Institute of Clinical Excellence
NIMP	Non Investigative Medicinal Product
NO	Nitrous Oxide
PIS	Patient Information Sheet
PMG	Project Management Group
RCOG	Royal College of Obstetricians and Gynaecologists
R&D	Research & Development
REC	Research Ethics Committee
RCT	Randomised Controlled Trial
RP	Retained placenta
SAE	Serious Adverse Event
SAR	Serious Adverse Reaction
SmPC	Summary of Product Characteristics
SOP	Standard Operating Procedure
SUSAR	Suspected Unexpected Serious Adverse Reaction
TMF	Trial Master File
TSC	Trial Steering Committee
UAR	Unexpected Adverse Reaction

SUMMARY

A retained placenta (RP) is a complication after a normal birth, which affects nearly 11,000 women in the UK a year. This is where the placenta is not delivered spontaneously after giving birth. It is a major cause of postpartum haemorrhage (major loss of blood) which can lead to the death of the mother. The recommended treatment for RP is a surgical procedure - manual removal of placenta (MROP). This is a painful and unpleasant intervention for the women, involving additional hospital stay, and is an expensive outcome for the NHS. It is widely recognised that non-surgical management options for RP are limited and it has been recommended that research is needed into new medical treatments for RP. New effective treatments for RP would dramatically reduce the number of women requiring MROP with the operation being restricted to the small minority of women with particularly stuck placentae. The reduction in operative interventions would have cost benefits for the NHS and also for women in terms of increased satisfaction, less separation of mother and baby immediately after birth, and reduced morbidity.

This study will try to prove the clinical and cost effectiveness of a known treatment for angina, Glyceryl trinitrate (GTN) used to treat RP. We will compare GTN against a placebo (dummy treatment) in a randomised controlled blinded trial (GOT-IT).

The GOT-IT Trial will be conducted in two phases. The first phase will involve an internal pilot study where the aim will be to test out and refine trial procedures in a small number of hospital sites. The second phase will be the main trial where recruitment will be extended to a larger number of hospitals in order to determine clinical and cost effectiveness.

Definition of 'Retained Placenta'

As per NICE guidelines, a retained placenta will be defined as a placenta which remains undelivered 30 minutes after the birth of the baby with active management of the third stage (comprising routine use of uterotonic drugs, early clamping and cutting of the cord and controlled cord traction) or 60 minutes with physiological management of the third stage (no routine use of uterotonic drugs, no clamping of the cord until pulsation has ceased and delivery of the placenta by maternal effort) followed by active management.

1 INTRODUCTION

1.1 BACKGROUND

A retained placenta (RP) is diagnosed when the placenta is not delivered within 30 minutes following active management, or 60 minutes following physiological and active management of the third stage of labour after delivery of the baby (NICE).[1] It affects 2% of vaginal deliveries[2] which equates to nearly 11,000 women in the UK per annum. RP is a major cause of postpartum haemorrhage[3, 4] with major obstetric haemorrhage itself affecting nearly 1 in 180 women and being the commonest cause of significant maternal morbidity (SCASM report 2012).[5] Following failure of active or physiological management, NICE recommends that RP should be treated by the operative procedure of manual removal of placenta (MROP).[1] The Cochrane Group,[6] NICE[1] and the Intrapartum Clinical Studies Group[7] all recognise that non-surgical management options for RP are limited and have recommended that research is needed into new medical strategies for RP. New (and effective) treatments for RP would dramatically reduce the number of women requiring MROP with the operation being restricted to the small minority of women with particularly adherent placentae (partial placenta accreta). The reduction in operative interventions would have cost benefits for the NHS and also for women in terms of increased satisfaction, less separation of mother and baby immediately after birth, and reduced morbidity.

1.2 RATIONALE FOR STUDY

1.2.1 Rationale for GTN (nitric oxide donor) for RP

The first report of a nitric oxide donor being used as a treatment for RP was in 1811 when inhalation amyl nitrate was used to treat a uterine constriction ring, thus facilitating MROP.[8] Since then, numerous observational studies have suggested that nitric oxide donors including GTN might be effective for the management of RP. Many of these studies have used intravenous boluses of GTN[9] ranging between 50–200 µg with uterine relaxation occurring about 60 seconds after injection and lasting for 2 minutes. Of the 5 observational studies which report the use of intravenous GTN (50mcg – 200mcg, up to two doses; n=87 women in total) for management of RP, success rates range from 94% - 100%[10-13] although with significant maternal side-effects including a dose dependant drop in systolic and diastolic blood pressure, rise in heart rate, sustained uterine relaxation and headache.[12, 13] Although in these small studies, intravenous GTN appears to be efficacious for RP, this route of administration is not practical in all settings and intravenous administration causes unacceptable side-effects including symptomatic hypotension at higher doses.[14]

More recently, sublingual administration has been trialed, with studies reporting experience with sublingual GTN tablet and spray. Using a sublingual GTN tablet, two small studies (n=36 women) suggest a beneficial effect of sublingual GTN tablet; however the third larger study does not (RCT ISRCTN34755982; 37.3% vs 20.4%; GTN vs placebo; $p > 0.05$; n=105 women).[15] All studies using sublingual GTN tablet have used 1mg. The alternative preparation for sublingual administration is GTN spray.[16] Compared to the tablet preparation, sublingual GTN spray has several advantages including stability at room temperature, significant reduction in latency of onset with onset beginning at 30-45 seconds, peaking at 90-120 seconds and lasting up to 5 minutes,[17-19] and fewer objective and subjective side-effects.[20] GTN spray is accepted obstetric management in other scenarios where rapid tocolysis is required such as uterine relaxation for release of a trapped head in breech delivery or at caesarean section.[16] Furthermore, anecdotal reports suggest that GTN spray may also have a use in management of RP.[16]

1.2.2 Biological plausibility of GTN for management of RP

It is likely that failure of myometrial contractions, placental trapping and adherence to the myometrium contribute variably to the ultimate clinical diagnosis of RP. In placentae which are detached but trapped behind a myometrial constriction ring, GTN could potentially treat RP simply by relaxing local uterine muscle constriction thereby effecting placental release.[6] For adherent placenta, Farley *et al* have suggested that nitric oxide mediated contraction and

relaxation of human chorionic villi along their longitudinal axis might serve as a GTN mediated mechanism for placental separation.[21] For placentae that are retained due to partial placenta accreta, currently available nitric oxide donor drugs (including GTN) are unlikely to effect release and surgical management is likely to remain the mainstay of treatment.

1.2.3 Summary

To summarise, although a growing body of evidence supports a use for GTN for treatment of RP, much of this evidence is based on anecdotal case-reports or clinical 'trials' which are non-randomised, do not include a placebo arm and are underpowered. Further, in the context of constrained maternity resources in a publicly funded health system, it is important to quantify the costs associated with the use of GTN (including any subsequent monitoring costs and costs associated with complications) in relation to its effectiveness and any subsequent cost savings it may deliver over standard practice. There is therefore an urgent need for a pragmatic clinical trial of GTN for RP to determine whether GTN is efficacious, safe, acceptable and cost-effective as a treatment for RP before a treatment which may (or may not) work is embedded within routine clinical practice. Our proposed randomised placebo controlled double blind pragmatic UK-wide GOT-IT trial RCT (with internal pilot study) will definitively determine whether sublingual GTN is (or is not) clinically and cost effective for management of RP.

2 STUDY OBJECTIVES

2.1 OBJECTIVES

The overall aim of the randomised placebo controlled double blind pragmatic UK-wide GOT-IT trial RCT (with internal pilot study) is to determine the clinical effectiveness and cost-effectiveness of sublingual GTN spray compared with placebo in reducing the need for MROP in women with RP after vaginal delivery following failure of current management. Outcomes will be measured over four inter-related domains – clinical, safety, patient-sided and economic.

2.1.1 Primary Objective

The primary research objectives of the **internal pilot RCT** are:

1) To demonstrate trial processes for approaching women, gaining consent, randomising, treating and assessing outcomes are optimal, and to implement improvements as required;
2) To determine achievable recruitment rates;
3) To determine the likely effect size, to inform a calculation on whether the planned sample size can be reduced whilst maintaining study power;
4) To pilot and modify if required the post-partum questionnaires (assessment of patient satisfaction and collection of health service use outcomes).

The primary research objectives of the **substantive GOT-IT RCT** are:

1) To determine the clinical effectiveness of sublingual GTN in treating RP and avoiding MROP in women with vaginal delivery following failure of current management (defined as a third stage of labour lasting more than 30 minutes after active management or 60 minutes after physiological followed by active management respectively) (clinical domain);
2) To determine the side-effect profile for GTN given to treat RP (safety domain);
3) To assess patient satisfaction with GTN given for RP (patient-sided domain);
4) To assess the net costs (or cost savings) to the National Health Service of using GTN for the treatment of RP compared to standard practice (economic domain).

2.1.2 Secondary Objectives

To assess NHS costs in relation to the primary outcome and range of secondary outcomes expected to differ between arms of the trial, using a cost-consequence balance sheet approach.

2.2 OUTCOMES

2.2.1 Primary Outcomes

Primary outcomes will be measured over four interrelated domains of clinical, safety, patient-sided and economic.

1) **Clinical**: need for MROP, defined as the placenta remaining undelivered 15 minutes post study treatment and/or being required within 15 minutes of treatment due to safety concerns.

2) **Safety**: measured blood loss between administration of treatment and transfer to the postnatal ward or other clinical area (e.g. labour ward high dependency).

3) **Patient-sided**: satisfaction with treatment and side effect profile assessed by questionnaire. It will be informed by our qualitative studies and based on questionnaires we have previously used to assess satisfaction.[22]

4) **Economic**: Net incremental costs (or cost savings) to the National Health Service of using GTN versus standard practice. Costs will include GTN (dose and time to administer drug, monitor woman and deliver the placenta if effective), MROP, and further health service resource use to six weeks postnatal (as measured by the health service resource use questionnaire).

2.2.2 Secondary Outcomes

Secondary outcomes will be measured over two interrelated domains of clinical and economic outcomes. The feasibility of the collection of secondary outcomes will be tested during the internal pilot phase.

1) **Clinical outcomes**:
i) Fall in haemoglobin of more than 15% between recruitment and the first postnatal day;
ii) time from randomisation to delivery of placenta;
iii) MROP in theatre;
iv) need for earlier than planned MROP on the basis of the clinical condition;
v) fall in systolic or diastolic blood pressure of more than 15mmHg and/or increase in pulse of more than 20 beats/minute between baseline and 5 and 15 minutes post-administration of active/placebo treatment;
vi) need for blood transfusion between time of delivery and discharge from hospital;
vii) need for general anaesthesia;
viii) maternal pyrexia (one or more temperature reading of more than 38˚C within 72hrs of delivery or discharge from hospital if discharge occurs sooner);
ix) sustained uterine relaxation after removal placenta requiring uterotonics;

2) **Costs**: The mean costs will be summarised by treatment allocation group, and the incremental cost (cost saving) associated with the use of GTN will be estimated using an appropriately specified general linear model. The cost data will be presented alongside the primary and secondary outcome data in a cost-consequence balance sheet, indicating which strategy each outcome favours.

There are no secondary patient-sided or safety outcomes.

3 STUDY DESIGN

3.1 OVERVIEW

We will undertake an internal pilot RCT followed by a substantive placebo controlled double blind pragmatic UK-wide trial (GOT-IT Trial) to determine the effectiveness of GTN for treating RP and avoiding MROP.

75 participants will be recruited from 8 centres in the internal pilot RCT.

The first meeting of the independent Data Monitoring Committee (DMC) will be organised before the internal pilot commences for the committee to adopt the protocol, accept their remit and agree the group sequential monitoring plan. We will then ask them to meet again at around 75 participants recruited (primarily from the 8 centres that start first as the internal pilot sites) and review recruitment and other outputs on trial processes from the internal pilot. The study Trial Steering Committee (TSC) will have met a couple of weeks prior to this and the report to the DMC will include the TSC's thoughts on the signals from the internal pilot. Assuming the DMC recommends progression, we will then proceed to rapidly expand the trial to the full set of sites, and proceed with the group sequential design which, if it recruits to maximum size, will recruit 1100 participants (maximum number randomised 1086, with the uplift to 1100 to account for a small allowance for withdrawal of consent or other rare loss to follow-up) across 20 sites over 42 months.

The primary outcomes will be measured over four interrelated domains (clinical, safety, patient-sided and economic) with secondary outcomes being measured over clinical and economic domains. The pre-specified group sequential monitoring plan will require the DMC to look at accumulating data on 5 occasions at regularly spaced intervals the first of which is scheduled for when 218 women have completed the study and have full data for analysis. The DMC will advise the TSC on adapting the trial design to either a) stop prematurely for futility (no prospect of establishing a treatment effect of at least 10%), b) stop prematurely if proof beyond a reasonable doubt is established that there is a convincing treatment benefit of at least 10%.

3.2 INTERNAL PILOT RCT

3.2.1 Detailed Design of Internal Pilot RCT

Ahead of the expansion to the full site list we will first undertake an internal pilot RCT specifically to provide reassurance on all the trial processes, including recruitment, consent, randomization, delivery of treatment, and follow-up assessments to ensure that all run smoothly. A nested qualitative study will be undertaken during the internal pilot RCT to adjust strategies to (a) maximise recruitment into the main trial; (b) optimise opportunities for getting informed consent; and, (c) ensure staff are given appropriate training and support to help promote the successful delivery of the main trial.

The internal pilot will run in 8 of the trial sites. The purpose of these pilot sites is to provide reassurance about all of the trial processes, including recruitment, consent, randomisation, delivery of the intervention, and measurement of the primary and secondary outcomes.

3.3 NESTED QUALITATIVE STUDY

3.3.1 Study Design

The nested qualitative study will be informed by the principles of grounded theory research[23] which entails simultaneous data collection and analysis, and allows the research questions and sampling to be revised in light of emerging findings, including any unanticipated issues which might arise during the implementation and delivery of the internal pilot RCT. In-depth interviews will be conducted with women invited to take part in the pilot RCT and staff members involved in the recruitment/consent process. These interviews will be informed by topic guides, which may be revised in light of emerging findings. The open-ended nature of the interviews will afford the flexibility necessary for research participants to raise and discuss issues which are salient to them, including those unforeseen at the outset of the pilot RCT.

3.3.2 Aims

To explore women's and staff's experiences of, and views about, the information and consent pathway used in the pilot RCT; to establish women's likes and dislikes of the interventions and procedures received in the pilot RCT.

3.3.3 Objectives

To refine/improve the information and consent pathway used in the substantive RCT to maximise recruitment and informed consent; to identify better ways of supporting staff involved in recruitment; to refine the questionnaire used to assess patient satisfaction with GTN given for RP.

3.3.4 Research Questions

The specific research questions which will be addressed by the qualitative studies are:
i) What are women's views about the timing of delivery and content of the information provided during the pilot RCT? In what ways do they think the information and consent pathway could be improved, and why?
ii) Why did women agree or decline to take part in the pilot RCT?
iii) Does the consent process give women a good understanding of the trial – if not how could this understanding be improved?
iv) What are women's likes and dislikes of the trial interventions and procedures?
v) What are staff member's experiences and views of recruiting women with RP into pilot RCT; how do they think the recruitment/consenting procedures might be improved; how, if at all, could they be better supported to undertake future recruitment?
vi) Do any unforeseen difficulties/issues arise during the pilot RCT; how might these be overcome in the substantive RCT?

3.3.5 Sample and Recruitment

Recruitment will take place in up to eight centres involved in the internal pilot RCT using an opt-in procedure. Approximately 25 women (comprising both decliners and those who took part in the internal pilot RCT) and 20 staff members will be selected for interviews. These sample sizes have been determined to allow a full range of themes and perspectives to be identified and explored in-depth and for data saturation to occur in key areas (saturation takes place when no new findings arise from an analysis of new data collected).[24, 25] We will initially recruit staff who are directly involved in the recruitment/consent process (i.e. obstetricians, labour ward midwives); however, if necessary, sampling may be broadened to include other staff members (e.g. anaesthetists) who may potentially also influence decisions about which women meet eligibility criteria and are approached to take part in the trial. We will also aim to interview staff who have experience of recruiting on day, night, and weekend shifts as it is possible that different issues may arise according to the time of day and which staff are on duty. If possible, women will be purposively sampled to include a diversity of occupational and educational backgrounds.

3.3.6 Data Collection

When possible, interviews with women will take place within four weeks of being approached to take part in the trial, to eliminate problems with recall bias. Staff will be approached and interviewed after they have had direct experience of attempting recruitment into the trial. All interviews will be undertaken face-to-face when possible (otherwise telephone interviews will be used), digitally recorded (with consent) and transcribed in full for in-depth analysis.

3.3.7 Data Analysis

Data analysis will be an iterative process which starts as soon as data collection begins (see above). The method of constant comparison will be used to identify issues and themes within and between interviews.[26] Team members will independently review interview data and regular meetings will be held to explore study participants' underlying reasoning, discuss deviant cases and reach agreement on recurrent themes and findings. Interviews will be coded to capture data relating to these themes and findings to allow for further in-depth analysis. QSR Nvivo 2, a qualitative data-indexing package, will be used to facilitate data coding and retrieval. A series of recommendations for refining the information and consent pathway (if necessary) and

providing support for staff involved in recruitment to the substantive RCT will be made in light of data analysis.

3.3.8 Implementation of Recommendations from Qualitative Research

An implementation group will be set up to enable fast and effective application of the qualitative findings. This group will comprise the CI (FD), other co-investigators (e.g. midwife S B-S), the Trial Manager, a patient representative (i.e. a women affected by RP) and a representative from one other recruiting centre. The implementation group will be convened prior to the data collection phase to help refine the interview topic guides and to discuss sampling strategies. Another face-to-face meeting will take place at the end of qualitative phase to enable the researchers to feedback their recommendations to the group to enable fast implementation into the main trial. If necessary, group members will be consulted on ad hoc basis during data collection, if their specific expertise is required.

3.4 SUBSTANTIVE RCT

3.4.1 Detailed Design of Substantive RCT

In GOT-IT, there is too much uncertainty in two crucial parameters to commit to a fixed sample size design. We believe that the most appropriate primary outcome is the proportion of women needing surgical intervention for removal of the placenta – this is a painful and unpleasant intervention for the women, involving additional hospital stay, and is an expensive outcome for the NHS. However, there is considerable uncertainty as to how many women who may be eligible for trial entry actually go on to require surgery due (a) to lack of knowledge of frequency of spontaneous delivery of the placenta beyond the timeframe when GTN has a pharmacological effect and (b) to variations in local clinical practice (e.g. logistics and time taken to organise theatre space and skilled staff to perform MROP). The routinely recorded statistics are not sufficiently detailed for these variables to be accurately determined. Second, we are very unsure as to what the magnitude of the benefit will be from GTN spray. To properly reflect these uncertainties, and give us a design which is flexible enough to maximise the chance that we efficiently detect and estimate the true benefit of treatment in the quickest time with the right number of participants, but equally to give ourselves the controlled opportunity to abandon the trial if it turns out that no worthwhile treatment effect actually exists (via so called futility analyses), we propose a **group sequential design.** We believe that for the situation of the GOT-IT trial, this is the ideal design because it enables us to present the maximum size of trial that is needed, alongside a flexible group sequential approach that allows the trial to terminate early for one of two scenarios. The first scenario is overwhelming evidence of benefit (due perhaps to a large treatment effect and/or less variability in the outcome measure). The second scenario is due to a suitably defined futility – that is, having got a certain way to the maximum trial size, we are confident that a large treatment effect is implausible, and that the current estimate of the treatment effect is sufficiently precise to be convincing, allowing the trial to terminate early.

We are able to state what the maximum size of trial is, because we are sure (from discussing with clinical colleagues and also taking soundings from mothers and mothers to be) that an absolute benefit of 10% would be the minimum to make it worth implementing this intervention in practice. From a statistical perspective we know the maximum variability in a binary outcome (need for surgical intervention – yes or no) occurs at a 50% rate in the placebo spray arm. On a fixed sample approach at 90% power and 5% level of significance, this would need 1038 women (519 in each group) to demonstrate a 10% change from 50% on placebo to 40% on GTN spray. Since the outcome is recorded within minutes of the intervention on the hospital systems (surgery took place yes/no), we anticipate minimal (if any) loss to follow up. So we can be confident that there is no need for a trial larger than this (except see below for the need for a small uplift to a possible maximum of 1086 women to allow for the multiple sequential evaluations of the data) to reliably answer this question – and it might be that a trial much smaller will deliver the answer in a statistically robust way.

There are very many options to decide upon for a group sequential design, and the DMC is central to its implementation and interpretation. As such we will have detailed discussions with the DMC, and in particular the independent statistician, over the detail of the group sequential design. It will be of critical importance that this DMC is comfortable and competent with such a

design. At this stage, we envisage using a Lan-DeMets alpha spending approach with O'Brien Fleming boundaries. We would specify this as a two sided test, with asymmetry for the efficacy and futility boundaries. We would propose five interim evaluations, equally spaced at 218, 434, 651, 868, and 1086 participants. There is fairly clear evidence that looking more than five times is not advantageous, statistically. We prefer the O'Brien-Fleming boundaries since these preserve much of the alpha for the later evaluations – there is the possibility of stopping early at the 218 and 434 looks, say, but only for very strong evidence (either way), and the advantage is that if one has to go to full trial size you have not compromised your ability to determine a treatment effect near the 5% level of significance by 'wasting the alpha' in pointless early evaluations. We have explored various options using SAS PROC SEQDESIGN and will share these with the DMC in our discussions to agree the specific group sequential approach we will adopt. They are all very similar in respect of maximum sample size and organising the DMC meeting dates. Figure 1 summarises the important aspects of the design.

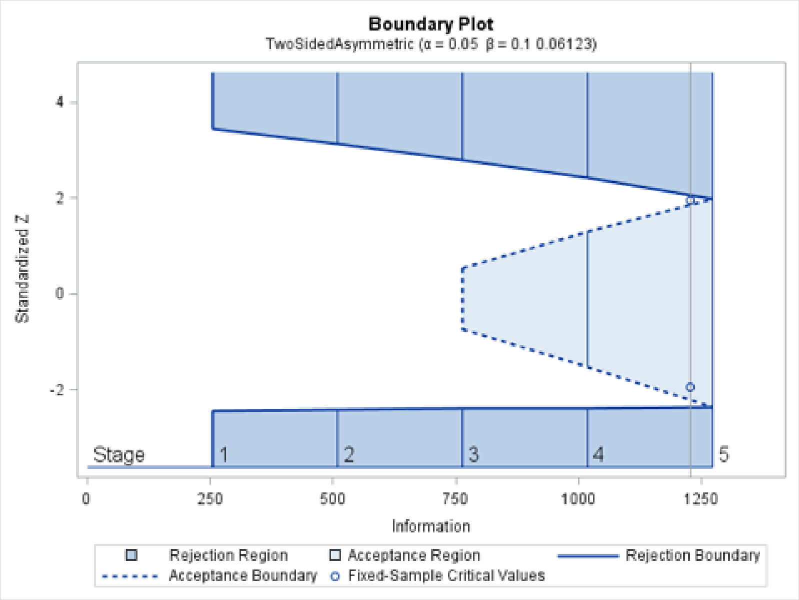

Figure 1: Group sequential design

3.4.2 Economic Evaluation

While the cost of GTN is low, there will still be costs associated with its administration and the monitoring and management of women thereafter. Should the intervention prove effective, it will be important in the context of scarce maternity resources to explicitly quantify the net costs (or cost savings) associated with its use. Provision is therefore being made to carry out a simple cost analysis using clinical and resource use data being collected for individual participants recruited to the trial. This analysis will explicitly quantify the difference in mean costs between the active intervention and placebo arm. Research costs associated with placebo delivery will be factored out of the analysis to estimate the incremental cost (or cost savings) of the active intervention versus standard practice.

Resource use associated with the alternative management strategies will be estimated from the time of randomisation through to 6 weeks post-partum. This will include: 1) staff time for administering the drug to patients; 2) resource use associated with any complications arising following administration of the study drug (e.g. blood pressure and/or heart rate monitoring); 3) subsequent costs associated with delivery of the placenta (either spontaneously or operatively); and 4) subsequent health service contact relating to retained products of conception up to 6 weeks post-partum.

The time from administration of the study drug to spontaneous delivery of the placenta (or the decision to proceed with manual removal), and the incidence of any complications following administration of the drug, will be collected via case report forms. Health service use in the six weeks following discharge will be collected via a small number of resource use questions included in the postnatal questionnaires. Resource use will be valued using routine sources of nationally relevant unit costs (Department of Health, 2012; PSSRU, 2012).

The mean costs will be summarised by treatment allocation group, and the incremental cost (cost saving) associated with the use of GTN will be estimated using an appropriately specified general linear model. The cost data will be presented alongside the primary and secondary outcome data in a cost-consequence balance sheet, indicating which strategy each outcome favours.

4 STUDY POPULATION

4.1 SETTING

The setting for the internal pilot RCT and substantive trial will be a minimum of 20 delivery wards in UK maternity hospitals. The delivery wards will be of varying size and location ensuring the results of the trial will be generalisable to the UK. Women do not have to have delivered in obstetric units to be eligible for trial entry i.e. if a woman develops a RP following failure of current management after giving birth at home or a stand-alone or along-side midwifery delivery unit, she will still be eligible for trial entry once admitted to one of the recruiting centres. However, although the referring setting may provide information about the study, consent, randomisation and administration of trial medication must always be undertaken in the obstetric (trial) unit.

All women with RP at risk of needing MROP after vaginal birth after failure of current management (defined as a third stage of labour lasting more than (i) 30 minutes after active management or (ii) 60 minutes following physiological followed by active management, respectively).

4.2 NUMBER OF PARTICIPANTS

4.2.1 Internal Pilot RCT

Number of sites : 8

Number of participants: around 75

4.2.2 Substantive RCT

Number of sites: Minimum of 20

Number of participants: The final number of participants for the full trial will be informed by the group sequential design. However, if the trial recruits to maximum size, it will recruit 1100 participants (maximum number randomised 1086, with the uplift to 1100 to account for a small allowance for withdrawal of consent or other rare loss to follow-up).

4.2.3 Projected Trial Duration

42 months

4.3 INCLUSION CRITERIA

- Women with retained placenta.
- Women aged 16 or over.
- Women with vaginal delivery (including women with a previous caesarean section).
- Haemodynamically stable (must satisfy all 3 definitions)
    - Haemodynamically stable.
    - Heart rate ≤119 bpm
    - Systolic blood pressure >100mmHg
- > 14 weeks gestation.

4.4 EXCLUSION CRITERIA

- Unable to give informed consent.
- Suspected placenta accreta/increta/percreta.
- Multiple pregnancy.
- Women having an instrumental vaginal delivery in theatre.
- Allergy or hypersensitivity to nitrates or any other constituent of the formulation.
- Taken alcohol in the last 24 hours.
- Concomitant use with phosphodiesterase inhibitors (such as sildenafil, tadalafil, or vardenafil).
- Contra-indication due to one of the following: Severe anaemia, constrictive pericarditis, extreme bradycardia, incipient glaucoma, Glucose-6-phosphatedehydrogenase-deficiency, cerebral haemorrhage and brain trauma, aortic and / or mitral stenosis and angina caused by hypertrophic obstructive cardiomyopathy. Circulatory collapse, cardiogenic shock and toxic pulmonary oedema.

Our trial design is pragmatic and our inclusion and exclusion criteria are therefore as broad and as inclusive as possible. We have however decided to exclude women with multiple pregnancies and women who are in theatre having an instrumental vaginal delivery for the following reasons:

1) Measured blood loss between randomisation and transfer to the postnatal ward is our pre-specified primary safety outcome. Multiple pregnancy is an independent risk factor for haemorrhage and these women are likely to have significantly higher blood loss than women with singleton pregnancies
2) If women are already in theatre having an instrumental vaginal delivery with adequate analgesia, then it is highly unlikely that the obstetrician delivering the baby will wait for 30 after active or 60 minutes after physiological followed by active management before diagnosing a RP. In this operative environment, in which skilled personnel and appropriate analgesia are already in place, the threshold to proceed to MROP and the adverse effects are much lower. Furthermore, our midwifery and lay representatives feel strongly that in this situation, it would be unethical and undignified for the woman to remain in theatre for longer than required for the sole purpose of fulfilling the eligibility criteria for the trial and,
3) Finally, if we were to include these two groups, we would need to stratify the data and significantly increase the sample size, which may make the trial unfeasible.

4.5 CO-ENROLMENT

Co-enrolment between CTIMP studies can be permitted providing there is a CTIMP-CTIMP agreement. The Sponsor requires proposals for co-enrolment between CTIMP studies to be captured in a written, authorised agreement between the Sponsors and Investigators of each study.

The participant will be allowed to co-enrol if they are currently taking part in a non-interventional trial.

5 PARTICIPANT SELECTION AND ENROLMENT

5.1 IDENTIFYING PARTICIPANTS

We will initially follow the RCOG best practice recommendations for recruitment and obtaining consent for intrapartum research studies.[27] These recommendations are based on the work by Vernon et al, [28] who developed a consent pathway for women with RP who were recruited to the RELEASE trial of umbilical vein oxytocin for RP.[29] In brief, where possible outline

information about the trial (including researcher contact details) will be made available to all women at antenatal booking. Trial posters will be displayed in antenatal clinics and labour wards at recruiting sites. If women wish more detailed information, this will be available in the form of patient-focused newsletters distributed to antenatal clinics and on labour wards, or by direction to the study website.

Screening logs will be completed at each recruiting site to document the number of potentially eligible women and the reasons for ineligibility or refusal to participate. Clinical teams at recruiting hospitals, working alongside the local research midwife, will be responsible for identifying participants for the trial. .

5.2 CONSENTING PARTICIPANTS

Once a diagnosis of RP is made, a delegated and trained member of the clinical/research team will discuss the trial and provide a summary information sheet. A more detailed Patient Information Sheet (PIS) will be given at the same time but as this is a time critical and potentially stressful clinical situation, a summary is more appropriate. Formal consent (written or verbal followed up by written at a later stage) will then be sought by a trained and delegated doctor, midwife or member of the research team.

5.3 SCREENING FOR ELIGIBILITY

Women with RP who fulfil the inclusion criteria and do not meet exclusion criteria will be eligible for study entry following informed consent. No additional pre-randomisation tests are required to confirm eligibility.

5.4 INELIGIBLE AND NON-RECRUITED PARTICIPANTS

Participants who are ineligible and/or who are not recruited to the trial will have standard treatment for management of RP which may include transfer to theatre for MROP.

No further information will be collected on women who are ineligible for recruitment.

5.5 RANDOMISATION

5.5.1 Randomisation Procedures

Randomisation packs will be ordered from pharmacy and kept on labour ward. Study drugs will be provided to site pharmacies in pre-packed randomised permuted blocks. Once a participant is recruited the study drug will be allocated by taking the next available treatment pack from the shelf. The label on the study drug package and the spray canister will be pre-printed with a study drug number. The investigator/practitioner will record the study drug number on the paper CRF. After demonstrating its use, the delegated practitioner will give the study drug to the women to self-administer. The study will be performed double masked so neither the patient nor the Investigator will know which treatment has been allocated. Breaking of the study masking will only be performed where knowledge of the treatment is absolutely necessary for safe management of the patient.

5.5.2 Treatment Allocation

Treatment allocation will be on a 1:1 ratio.

5.5.3 Emergency Unblinding Procedures

Central unblinding procedures will be maintained by CHaRT who will hold the randomisation list for the trial (study drug pack numbers and allocation). Unblinding (emergency or otherwise) can be carried out by a senior clinician (normally a consultant). The senior clinician will call an Interactive Voice Response System (IVRS) and enter their name, the reason for unblinding, and the study drug number into the automated system. This phone line will be available at all times. The unblinding will be recorded on the trial database and the trial manager will be informed. Unless there is a clinical requirement, the masking will not be broken until after data

entry is complete, the validity of the data is checked, all queries resolved and the patient populations agreed.

5.5.4 Withdrawal of Study Participants

Participation in the study is voluntary. A patient has the right to completely withdraw from the study at any time for any reason.

Given the nature of the intervention (i.e. administration of intervention/placebo spray at a single time-point), unless the participant withdraws from the study between consent and administration of the IMP, it is likely that the intervention will have been given prior to participant withdrawal. The reason, duration and circumstances for premature discontinuation will be documented in the CRF.

6 INVESTIGATIONAL MEDICINAL PRODUCT AND PLACEBO

6.1 STUDY DRUG

Sublingual GTN is maximally effective in causing uterine and cervical relaxation by 5 minutes post administration. Thus, if spontaneous delivery of the placenta is going to occur, we would expect this to happen around 5 minutes after drug administration. If spontaneous placental delivery does not occur, a further attempt to deliver the placenta by controlled cord traction will be made. No further study drug treatment (GTN or placebo). If the placenta remains undelivered 15 minutes after administration of the treatment we think that it is highly unlikely that it will deliver spontaneously thereafter – we consider that any further delay risks haemorrhage. Thus the decision that MROP is needed (primary clinical outcome) will be made and the participant will be transferred to theatre for the definitive management of MROP as soon as possible. Additionally, if there are clinically significant side-effects (e.g. haemorrhage, symptomatic maternal hypotension and/or tachycardia) before 15 minutes have elapsed after GTN/placebo treatment; the participant will be transferred to theatre for immediate MROP. No further information will be collected on women with RP who are ineligible or not recruited because they do not fulfil the inclusion/exclusion criteria, others than the number of such women for inclusion in trial metrics.

6.1.1 Study Drug Identification

Nitrolingual Pump Spray [Coro-Nitro]

A liquid within non-pressurised, red plastic-coated glass bottle fitted with a pump capable of delivering a metered dose containing 400μg of glyceryl trinitrate.

Excipients: The formulation contains fractionated coconut oil, absolute ethanol, medium chain partial glycerides and peppermint oil.

6.1.2 Study Drug Manufacturer

Pharmasol Limited.
North Way, Andover, Herts. SP10 5AZ, UK.

6.1.3 Marketing Authorisation Holder

Product License Holder:
Ayrton Saunders Ltd.
Reeds Lane, Moreton, Wirral, CH46 1DW, UK.

Distributed by:
Winthrop Pharmaceuticals Winthrop, PL16431/0018.
PO Box 611, Guildford, Surrey, GU1 4YS, UK.

Marketing Authorisation number: PL 16431/0018

6.1.4 Labelling and Packaging

The GTN and placebo will be labelled appropriately by Sharp Clinical Services (UK) Ltd.

6.1.5 Storage

Study drugs will be prepared and kept on the delivery suite in pre-packed boxes. Study drugs will be stored at or below 30°C and have a shelf-life of 3 years.

Drugs will be kept in pharmacy with a small supply available in labour wards.

6.1.6 Summary of Product Characteristics

Information on the Summary of Product Characteristics (SmPC) is given in Appendix 1.

6.2 PLACEBO

Matched bottles will be supplied and labelled by Sharp. The bottles will be packaged in a study treatment pack and flag-labels will be applied.

6.3 DOSING REGIME

The sublingual intervention (experimental (GTN or control (placebo) spray) will be self-administered as a single intervention (two puffs (800µg active drug or placebo), no repeats)) as soon as possible after diagnosis of RP.

6.4 DOSE CHANGES

The treatment (2 puffs experimental (GTN) or control (placebo) spray) will be self-administered as a single intervention. No second intervention will be given.

6.5 PARTICIPANT COMPLIANCE

Participants will be taught and then observed self-administering the drug in an obstetric unit. We therefore expect that compliance will be high.

Any concerns about administration of the study drug or participant non-compliance will be recorded on the CRF and the reasons for non-compliance documented.

6.6 OVERDOSE

The risk of overdose will be low. Participants will be observed taking the study medication. In the unlikely event that an overdose has occurred or is suspected, the participant will be in hospital with direct access to medical care and observation.

However, the short half-life of the active intervention (GTN – duration) means that the duration of any side-effect secondary to overdose of the study medication will be transient.

GTN can cause hypotension, tachycardia, flushing, dizziness and headache in doses up to 800µg. Participants will have intravenous access secured prior to administration of the study medication. If symptomatic hypotension and tachycardia occur, these will be treated with fluid resuscitation and/or medication if required. Headache will be treated with analgesia only if prolonged and symptomatic.

6.7 OTHER MEDICATIONS

6.7.1 Non-Investigational Medicinal Products

Not relevant.

6.7.2 Permitted Medications

Permitted medications will comprise drugs which are used in standard care for postpartum women and/or management of RP. The table below is not a comprehensive list.

Class of allowable (with restrictions)		Examples (not comprehensive list)	Allowable treatment dose
Medication used for pain relief	Analgesics	Oral paracetamol (acetaminophen)	Oral paracetamol (acetaminophen)<br>Maximum dose of paracetamol or any paracetamol-containing products ≤4 g/day (8x500 mg/day)
	Anti-inflammatory	Aspirin<br>NSAID ibuprofen, diclofenac,<br>Stable inhaled corticosteroids	Per product label
	Opiods	Oxycodone<br>Codeine<br>Diamorphine<br>Dihydrocodeine<br>Morphine<br>Methadone<br>Tramadol<br>Fentanyl	As per product label
	Non-pharmacological therapies	Physical therapy, massage, aromatherapy	
	Local anaesthetics	Lidocaine<br>Bupivicaine<br>Levobupivicaine<br>Prilocaine<br>Tetracaine	As per product label
Medical used for control of nausea/vomiting	Anti-emetics	Cyclizine<br>Prochlorperazine<br>Promethazine<br>Metoclopramide<br>Ondansetron	As per product label

Medication used for uterine contractions		Oxytocin<br>Ergometrine maleate<br>Syntometrine<br>Misoprostol<br>Carboprost	

6.7.3 Prohibited Medications

Concomitant administration of phosphodiesterase inhibitors used for the treatment of pulmonary arterial hypertension (e.g. sildenafil, tadalafil, vardenafil, avanafil and udenafil)A severe and possibly dangerous fall in blood pressure may occur. This can result in collapse, unconsciousness and paradoxical myocardial ischaemia and may be fatal. Such use is therefore contra-indicated.

6.7.4 Recording Concomitant Medications

Use of all concomitant medications will be recorded from 24 hours prior to signing of the informed consent form until 24 hours after administration of study drug. Information relating to IV fluids is not required.

Use of analgesic or antibiotic medications will be recorded from 24 hours prior to signing of informed consent until 6 weeks after administration of study drug.

7 STUDY ASSESSMENTS

7.1 SAFETY ASSESSMENTS

- Medical history
- Concomitant medications
- Full blood count
- Blood pressure measured at Baseline, 5 and 15 minutes post-administration
- Pulse
- Temperature
- Blood loss
- Adverse events

7.2 STUDY ASSESSMENTS

Pre-randomisation:

- Screening
- Eligibility confirmed (including brief medical history and concomitant medication check)
- Informed consent
- Baseline observations (BP, pulse, temperature)
- Full blood count taken

Randomisation:
Treatment allocated and administered.

5 minutes:

- Observations (BP, pulse, temperature)

15 minutes:

- Observations (BP, pulse, temperature)
- Need for MROP

Before transfer:

- Blood loss measured

Before discharge:

- Full blood count taken
- Patient rated side-effects
- Patient rated satisfaction
- Adverse events recorded

Six weeks:

- Postnatal questionnaire (patient rated satisfaction, side effects and health resource use) posted back.
- Adverse events recorded

The primary clinical outcome (need for MROP, defined as the placenta remaining undelivered 15 minutes post treatment and/or being required within 15 minutes of treatment due to safety concerns) will be collected 15 minutes post administration of the study drug. The primary safety outcome (measured blood loss between administration treatment and transfer to the postnatal ward/other clinical area, e.g. labour ward high dependency) will be recorded immediately prior to transfer. The primary patient satisfaction outcome will be collected at six weeks by a self-completed satisfaction questionnaire and the primary economic outcome will be imputed from costs incurred at initial management of RP and management of further complications and self-reported health service usage and side-effects recorded from the postnatal patient questionnaires.

8 DATA COLLECTION

Outcome data up to the point of discharge will be collected locally by the clinical teams and local Research Midwife. Core data will be captured via a single source sheet, and along with additional data will be entered directly into electronic CRFs, developed by CHaRT, by the local Research Midwife at each centre. The postnatal paper-based questionnaires will be completed by participants at home and returned to the central trial office, and again will be inputted into an electronic database. The trial administrator and trial manager based in Edinburgh will liaise with local sites and CHaRT about data completeness and data queries. Missing data will be collected and fed-back from study centres by the local Research Midwifery Champion.

9 STATISTICS AND DATA ANALYSIS

9.1 SAMPLE SIZE CALCULATION

We believe that an absolute benefit of an increase in 10% of women avoiding the need for manual removal of the placenta will be sufficient advantage to change clinical practice and result in the widespread uptake of this simple, safe and cheap drug intervention (2 puffs (800µg) sublingual GTN spray administered within minutes of a diagnosis of suspected retained

placenta). Due to the sparse nature of poor quality data for the use of GTN for RP, treatment and control effect rate, there is at present considerably uncertainty in designing a definite trial. As well as including an internal pilot RCT, with a view to including those randomised in the final analysis, we believe that a sequential design is appropriate given the lack of evidence currently available. This will give the study an opportunity to stop early – via the full involvement of a DMC - if either the treatment effect is much larger than anticipated, or if conversely the treatment effect is sufficiently small as to be not worthwhile. We think we will need to randomise no more than 1086 participants (assuming a 50% control rate and a 10% absolute treatment effect, at 90% power and 5% level of significance). The outcome of each individual patient is known on the day of treatment, and so in effect, the data mature for this study in real time.

9.2 PROPOSED ANALYSES

The statistical analyses will be according to the Intention-To-Treat principle and will be governed by a comprehensive Statistical Analysis Plan which will be finalised before the data are unblinded for the final analysis. The interim analyses for the DMC will be specified within their DMC Charter, and results of these interim analyses will be in strict confidence (no member of the research group apart from the study statistician will be aware of the contents of these analyses). Advice on the final sample size required will, of course, be revealed to the TSC. The primary outcomes (clinical – manual removal of placenta; safety – blood loss; patient – satisfaction and economic – NHS costs) will be analysed using generalised linear models appropriate to the distribution of each outcome. These models will adjust for relevant baseline factors, and sensitivity analyses will assess the robustness of the findings to any missing data (for the primary clinical outcome this is expected to be virtually nil, as the outcome is measured within minutes of taking the intervention). Subgroup analyses of the primary outcome will be explored.

10 ADVERSE EVENTS

The Investigator is responsible for the detection and documentation of events meeting the criteria and definitions detailed below.

Full details of contraindications and side effects that have been reported following administration of the IMP can be found in the SmPC (Appendix 1).

Participants will be instructed to contact their Investigator at any time after consenting to join the trial if any symptoms develop. All adverse events (AE) that occur after joining the trial must be reported in detail in the AE form. In the case of an AE, the Investigator should initiate the appropriate treatment according to their medical judgment. Participants with AEs present up until the 6 week postnatal outcome assessment point must be followed up until resolution of the event.

10.1 DEFINITIONS

An **adverse event** (AE) is any untoward medical occurrence in a clinical trial participant which does not necessarily have a causal relationship with an investigational medicinal product (IMP).

An **adverse reaction** (AR) is any untoward and unintended response to an IMP which is related to any dose administered to that participant.

A **serious adverse event** (SAE), **serious adverse reaction** (SAR) is any AE or AR that at any dose:

- results in death of the clinical trial participant;
- is life threatening*;
- requires in-patient hospitalisation^ or prolongation of existing hospitalisation;
- results in persistent or significant disability or incapacity;
- consists of a congenital anomaly or birth defect;
- results in any other significant medical event not meeting the criteria above.

*Life-threatening in the definition of an SAE or SAR refers to an event where the participant was at risk of death at the time of the event. It does not refer to an event which hypothetically might have caused death if it were more severe.

^Any hospitalisation that was planned prior to randomisation will not meet SAE criteria. Any hospitalisation that is planned post-randomisation will meet the SAE criteria.

A suspected unexpected serious adverse reaction (SUSAR) is any AR that is classified as serious and is suspected to be caused by the IMP, that it is not consistent with the information about the IMP in the SmPC.

10.2 IDENTIFYING AEs AND SAEs

All AEs and SAEs will be recorded from the time a participant signs the consent form to take part in the study until the 6 week postnatal outcome assessment point. The half-life of GTN is very short and any adverse events will happen within the first 15 minutes after administration.

Participants will be asked about the occurrence of AEs/SAEs prior to discharge from the hospital and in the 6 week postnatal questionnaire. Open-ended and non-leading verbal questioning of the participant will be used to enquire about AE/SAE occurrence at discharge. The 6 week postnatal questionnaire will also ask participants if they have seen their GP, been admitted to hospital, or been prescribed any medication. If there is any doubt as to whether a clinical observation is an AE, the event will be recorded.

AEs and SAEs may also be identified via information from support departments e.g. laboratories.

10.3 RECORDING AEs AND SAEs

When an AE/SAE occurs, it is the responsibility of the Investigator to review all documentation (e.g. hospital notes, laboratory and diagnostic reports) related to the event. The Investigator will then record all relevant information on the SAE form (if the AE meets the criteria of serious).

Information to be collected includes type of event, onset date, Investigator assessment of severity and causality, date of resolution as well as treatment required, investigations needed and outcome.

In this study, the following events are expected and therefore they will not be reported to the Co-Sponsors. The clinician will assess **ALL** reported SAEs, confirm any that are ‘expected’, as detailed in the list below and will not be reported to the Co-Sponsors as an SAE **but** will be recorded in the CRF as a hospitalisation or outcomes and presented to the DMC, as part of the ongoing safety review.

For this study the following events are **NOT** considered SAEs:

i) Pregnancy is not considered an AE or SAE, as it is part of the inclusion criteria.

ii) Hospitalisations for treatment planned prior to randomisation and hospitalisation for elective treatment of a pre-existing condition will not be considered as an SAE. This includes pregnancy.

iii) Fall in haemoglobin of more than 15% between recruitment and the first postnatal day;

iv) MROP in theatre

v) need for earlier than planned MROP on the basis of the clinical condition,

vi) fall in systolic or diastolic blood pressure of more than 15mmHg and/or increase in pulse of more than 20 beats/minute between baseline and 5 and 15 minutes post-administration of active/placebo treatment;

vii) need for blood transfusion between time of delivery and discharge from hospital;

viii) need for general anaesthesia;

ix) maternal pyrexia (one or more temperature reading of more than 38^{0}C within 72hrs of delivery or discharge from hospital if discharge occurs sooner);

x) sustained uterine relaxation after removal of placenta requiring uterotonics.

10.4 ASSESSMENT OF AEs AND SAEs

Seriousness, causality, severity and expectedness of each AE will be assessed by the Principal Investigator. AEs will be assessed as though the participant is taking active IMP. Cases that are considered serious, possibly, probably or definitely related to IMP and unexpected (i.e. SUSARs) will be unblinded.

The Chief Investigator (CI) may not downgrade an event that has been assessed by an Investigator as an SAE or SUSAR, but can upgrade an AE to an SAE, SAR or SUSAR if appropriate.

10.4.1 Assessment of Seriousness

The Investigator will make an assessment of seriousness as defined in Section 10.1.

10.4.2 Assessment of Causality

The Investigator will make an assessment of whether the AE/SAE is likely to be related to the IMP according to the definitions below.

- Unrelated: where an event is not considered to be related to the IMP.
- Possibly Related: The nature of the event, the underlying medical condition, concomitant medication or temporal relationship make it possible that the AE has a causal relationship to the study drug. The assessment of causality will be made against the reference safety information found in the SmPC (Appendix 1).

Where non Investigational Medicinal Products (NIMPs) e.g. rescue/escape drugs are given: if the AE is considered to be related to an interaction between the IMP and the NIMP, or where the AE might be linked to either the IMP or the NIMP but cannot be clearly attributed to either one of these, the event will be considered as an AR. Alternative causes such as natural history of the underlying disease, other risk factors and the temporal relationship of the event to the treatment should be considered and investigated. The blind should not be broken for the purpose of making this assessment.

10.4.3 Assessment of Expectedness

If an event is judged to be an AR, the evaluation of expectedness will be made based on knowledge of the reaction and the relevant product information documented in the SmPC.

The event may be classed as either:

Expected: the AR is consistent with the toxicity of the IMP listed in the SmPC.

Unexpected: the AR is not consistent with the toxicity in the SmPC.

10.4.4 Assessment of Severity

The Investigator will make an assessment of severity for each AE/SAE and record this on the SAE form according to one of the following categories:

Mild: an event that is easily tolerated by the participant, causing minimal discomfort and not interfering with every day activities.

Moderate: an event that is sufficiently discomforting to interfere with normal everyday activities.

Severe: an event that prevents normal everyday activities.

Note: the term 'severe', used to describe the intensity, should not be confused with 'serious' which is a regulatory definition based on participant/event outcome or action criteria. For

example, a headache may be severe but not serious, while a minor stroke is serious but may not be severe.

10.5 REPORTING OF SAEs/SARs/SUSARs

Once the Investigator becomes aware that an SAE has occurred in a study participant, the information will be reported to the ACCORD Research Governance & QA Office **immediately or within 24 hours**. If the Investigator does not have all information regarding an SAE, they should not wait for this additional information before notifying ACCORD. The SAE report form can be updated when the additional information is received.

The SAE report will provide an assessment of causality and expectedness at the time of the initial report to ACCORD according to Sections 10.4.2, Assessment of Causality and 10.4.4, Assessment of Expectedness.

The SAE form will be transmitted by fax to ACCORD on **+44 (0)131 242 9447** or by email to safety.accord@ed.ac.uk or electronically via the trial website, or may be transmitted by hand to the office. Only forms in a pdf format will be accepted by ACCORD via email.

Where missing information has not been sent to ACCORD after an initial report, ACCORD will contact the Investigator and request the missing information until this is supplied.

All reports sent to ACCORD and any follow up information will be retained by the Investigator in the Investigator Site File (ISF).

10.6 REGULATORY REPORTING REQUIREMENTS

The ACCORD Research Governance & QA Office is responsible for pharmacovigilance reporting on behalf of the co-sponsors (Edinburgh University and NHS Lothian).

The ACCORD Research Governance & QA Office has a legal responsibility to notify the regulatory competent authority and relevant ethics committee (Research Ethics Committee (REC) that approved the trial). Fatal or life threatening SUSARs will be reported no later than 7 calendar days and all other SUSARs will be reported no later than 15 calendar days after ACCORD is first aware of the reaction.

ACCORD will inform Investigators at participating sites of all SUSARs and any other arising safety information.

An Annual Safety Report/Development Safety Update Report will be submitted, by ACCORD, to the regulatory authorities and RECs listing all SARs and SUSARs.

10.7 FOLLOW UP PROCEDURES

After initially recording an AE or recording and reporting an SAE, the Investigator will follow each participant until resolution or death of the participant. Follow up information on an SAE will be reported to the ACCORD office.

AEs still present in participants at the 6 week postnatal outcome assessment point will be monitored until resolution of the event or until no longer medically indicated.

11 PREGNANCY

Pregnancy is not considered an AE or SAE and it is very unlikely that a participant would become pregnant by the 6 week postnatal outcome assessment point (endpoint for the trial).

In the unlikely event that a participant becomes pregnant, the Investigator will collect pregnancy information, record the information on a Pregnancy Notification Form and submit this to the ACCORD office within 14 days of being made aware of the pregnancy.

All pregnant female participants will be followed up until following the outcome of the pregnancy.

12 TRIAL MANAGEMENT AND OVERSIGHT ARRANGEMENTS

12.1 PROJECT MANAGEMENT GROUP

The trial will be coordinated by a Project Management Group (PMG), consisting of the grant holders (Chief Investigator and Co-applicants), Trial Manager, Trial Administrator, representative from CHaRT (Trials Unit and Data Centre), representative from ACCORD (Sponsor) and a pharmacist.

Members of the PMG (or a delegated deputy) will be invited to attend the Trial Steering Committee meetings.

The Trial Manager will oversee the study and will be accountable to the Chief Investigator. CHaRT and the Trial Manager will be responsible for checking the CRFs for completeness, plausibility and consistency. Any queries will be resolved by the site Principle Investigator or delegated member of the trial team.

A Delegation Log will be prepared for each site, detailing the responsibilities of each member of staff working on the trial.

12.2 TRIAL STEERING COMMITTEE

A Trial Steering Committee (TSC) will be established to oversee the conduct and progress of the trial. The TSC will adhere to the terms of reference and reporting guidelines stipulated by the trial funding body (HTA). The TSC will meet regularly throughout the study and will liaise closely with the DMC and PMG.

12.3 DATA MONITORING COMMITTEE

An independent Data Monitoring Committee (DMC) will be established to oversee the safety of participants in the trial. The DMC will adhere to the terms of reference and the reporting guidelines stipulated by the trial funding body (HTA). The DMC will meet regularly throughout the study and will liaise closely with the TSC and PMG. Co-ordinated triggered meetings will take place at the end of the pilot study phase to consider the signals on trial progress from the internal pilot. This is distinct from the first scheduled formal interim analysis by the DMC which is due at 218 recruits having mature data.

12.4 INSPECTION OF RECORDS

Investigators and institutions involved in the study will permit trial related monitoring and audits on behalf of the sponsor, REC review, and regulatory inspection(s). In the event of an audit or monitoring, the Investigator agrees to allow the representatives of the sponsor direct access to all study records and source documentation. In the event of regulatory inspection, the Investigator agrees to allow inspectors direct access to all study records and source documentation.

12.5 RISK ASSESSMENT

An independent risk assessment will be performed by an ACCORD Clinical Trials Monitor to determine if monitoring is required and if so, at what level. An independent risk assessment will also be carried out by the ACCORD Quality Assurance Group to determine if an audit should be performed before/during/after the study and if so, at what locations and at what frequency.

12.6 STUDY MONITORING AND AUDIT

An ACCORD Clinical Trials Monitor or an appointed monitor will visit the Investigator site prior to the start of the study and during the course of the study if required, in accordance with the monitoring plan if required. Risk assessment will determine if audit, by the ACCORD QA group, is required. Details will be captured in an audit plan. Audit of Investigator sites, study management activities and study collaborative units, facilities and 3rd parties may be performed.

13 GOOD CLINICAL PRACTICE

13.1 ETHICAL CONDUCT

The study will be conducted in accordance with the principles of the International Conference on Harmonisation Tripartite Guideline for Good Clinical Practice (ICH GCP).

A favorable ethical opinion will be obtained from the appropriate REC and local R&D approval will be obtained prior to commencement of the study.

13.2 REGULATORY COMPLIANCE

The study will not commence until a Clinical Trial Authorisation (CTA) is obtained from the appropriate Regulatory Authority. The protocol and study conduct will comply with the Medicines for Human Use (Clinical Trials) Regulations 2004, as amended.

13.3 INVESTIGATOR RESPONSIBILITIES

The Investigator is responsible for the overall conduct of the study at the site and compliance with the protocol and any protocol amendments. In accordance with the principles of ICH GCP, the following areas listed in this section are also the responsibility of the Investigator. Responsibilities may be delegated to an appropriate member of study site staff.

13.3.1 Informed Consent

The Investigator is responsible for ensuring informed consent is obtained before any protocol specific procedures are carried out. The decision of a participant to participate in clinical research is voluntary and should be based on a clear understanding of what is involved. Due to the clinical situation, verbal consent may be provided but must be then followed by written consent.

Participants must receive adequate oral and written information – appropriate Participant Information and Informed Consent Forms will be provided. The oral explanation to the participant will be performed by the Investigator or qualified delegated person, and must cover all the elements specified in the Participant Information Sheet and Consent Form.

The participant must be given every opportunity to clarify any points they do not understand and, if necessary, ask for more information. The participant must be given sufficient time to consider the information provided. It should be emphasised that the participant may withdraw their consent to participate at any time without loss of benefits to which they otherwise would be entitled.

The participant will be informed and agree to their medical records being inspected by regulatory authorities and representatives of the sponsor(s) but understand that their name will not be disclosed outside the hospital.

The Investigator or delegated member of the trial team and the participant will sign and date the Informed Consent Form(s) to confirm that consent has been obtained. The participant will receive a copy of this document and a copy filed in the Investigator Site File (ISF) and participant's medical notes.

13.3.2 Study Site Staff

The Investigator must be familiar with the IMP, protocol and the study requirements. It is the Investigator's responsibility to ensure that all staff assisting with the study are adequately informed about the IMP, protocol and their trial related duties.

13.3.3 Data Recording

The Principle Investigator is responsible for the quality of the data recorded in the CRF at each Investigator Site. The source data plan identifies which source data correspond to CRF data and states which data are recorded directly into the CRF.

13.3.4 Investigator Documentation

Prior to beginning the study, each Investigator will be asked to provide particular essential documents to the ACCORD Research Governance & QA Office, including but not limited to:

- An original signed Investigator's Declaration (as part of the Clinical Trial Agreement documents);
- Curriculum vitae (CV) signed and dated by the Investigator indicating that it is accurate and current.

The ACCORD Research Governance & QA Office will ensure all other documents required by ICH GCP are retained in a Trial Master File (TMF), where required, and that appropriate documentation is available in local ISFs.

13.3.5 GCP Training

All study staff must hold evidence of appropriate GCP training.

13.3.6 Confidentiality

All laboratory specimens, evaluation forms, reports, and other records must be identified in a manner designed to maintain participant confidentiality. All records must be kept in a secure storage area with limited access. Clinical information will not be released without the written permission of the participant. The Investigator and study site staff involved with this study may not disclose or use for any purpose other than performance of the study, any data, record, or other unpublished, confidential information disclosed to those individuals for the purpose of the study. Prior written agreement from the sponsor or its designee must be obtained for the disclosure of any said confidential information to other parties.

13.3.7 Data Protection

All Investigators and study site staff involved with this study must comply with the requirements of the Data Protection Act 1998 with regard to the collection, storage, processing and disclosure of personal information and will uphold the Act's core principles. Access to collated participant data will be restricted to those clinicians treating the participants, representatives of the sponsor(s) and representatives of regulatory authorities.

Computers used to collate the data will have limited access measures via user names and passwords.

Published results will not contain any personal data that could allow identification of individual participants.

14 STUDY CONDUCT RESPONSIBILITIES

14.1 PROTOCOL AMENDMENTS

Any changes in research activity, except those necessary to remove an apparent, immediate hazard to the participant in the case of a urgent safety measure, must be reviewed and approved by the Chief Investigator.

Amendments to the protocol must be submitted in writing to the appropriate REC, Regulatory Authority and local R&D for approval prior to participants being enrolled into an amended protocol.

14.2 PROTOCOL VIOLATIONS AND DEVIATIONS

Prospective protocol deviations, i.e. protocol waivers, will not be approved by the sponsors and therefore will not be implemented, except where necessary to eliminate an immediate hazard

to study participants. If this necessitates a subsequent protocol amendment, this should be submitted to the REC, Regulatory Authority and local R&D for review and approval if appropriate.

Protocol deviations will be recorded in a protocol deviation log and logs will be submitted to the sponsors every 3 months. Each protocol violation will be reported to the sponsor within 24 hours of becoming aware of the violation.

14.3 SERIOUS BREACH REQUIREMENTS

A serious breach is a breach which is likely to effect to a significant degree:

(a) the safety or physical or mental integrity of the participants of the trial; or

(b) the scientific value of the trial.

If a potential serious breach is identified by the Chief Investigator, Principal Investigator or delegates, the co-sponsors (accord.seriousbreach@ed.ac.uk) must be notified within 24 hours. It is the responsibility of the co-sponsors to assess the impact of the breach on the scientific value of the trial, to determine whether the incident constitutes a serious breach and report to regulatory authorities and research ethics committees as necessary.

14.4 STUDY RECORD RETENTION

All study documentation will be kept for a minimum of 5 years from the protocol defined end of study point. When the minimum retention period has elapsed, study documentation will not be destroyed without permission from the sponsor.

14.5 END OF STUDY

The end of study is defined as the last participant’s last visit. For the GOT-IT Trial, this will be the return of the 6 week postnatal questionnaire for the last participant.

The Investigators and/or the TSC and/or the co-sponsor(s) have the right at any time to terminate the study for clinical or administrative reasons.

The end of the study will be reported to the REC and Regulatory Authority within 90 days, or 15 days if the study is terminated prematurely. The Investigators will inform participants of the premature study closure and ensure that the appropriate follow up is arranged for all participants involved.

A summary report of the study will be provided to the REC and Regulatory Authority within 1 year of the end of the study.

14.6 CONTINUATION OF DRUG FOLLOWING THE END OF STUDY

The study drug is being given for the management of RP. There is no indication for ongoing treatment for RP because the placenta will be surgically or non-surgically delivered.

14.7 INSURANCE AND INDEMNITY

The co-sponsors are responsible for ensuring proper provision has been made for insurance or indemnity to cover their liability and the liability of the Chief Investigator and staff.

The following arrangements are in place to fulfil the co-sponsors' responsibilities:

- The Protocol has been designed by the Chief Investigator and researchers employed by the University and collaborators. The University has insurance in place (which includes no-fault compensation) for negligent harm caused by poor protocol design by the Chief Investigator and researchers employed by the University.
- Sites participating in the study will be liable for clinical negligence and other negligent harm to individuals taking part in the study and covered by the duty of care owed to them by the sites concerned. The co-sponsors require individual sites participating

in the study to arrange for their own insurance or indemnity in respect of these liabilities.

- Sites which are part of the United Kingdom's Nation Health Service will have the benefit of NHS Indemnity.
- Sites out with the United Kingdom will be responsible for arranging their own indemnity or insurance for their participation in the study, as well as for compliance with local law applicable to their participation in the study.
- The manufacturer supplying IMP has accepted limited liability related to the manufacturing and original packaging of the study drug and to the losses, damages, claims or liabilities incurred by study participants based on known or unknown Adverse Events which arise out of the manufacturing and original packaging of the study drug, but not where there is any modification to the study drug (including without limitation re-packaging and blinding).

15 REPORTING, PUBLICATIONS AND NOTIFICATION OF RESULTS

15.1 AUTHORSHIP POLICY

Ownership of the data arising from this study resides with the study team. On completion of the study, the study data will be analysed and tabulated, and a clinical study report will be prepared in accordance with ICH guidelines.

For the final clinical study report arising from the GOT-IT trial, the manuscript will be drafted by the Chief Investigator, who will be the corresponding author. As according to the SPIRIT guidelines (http://www.spirit-statement.org/31b-authorship/), substantive contributions to the design, conduct, interpretation, and reporting of the trial will be recognised through the granting of authorship on the final trial report. Those fulfilling authorship will be listed by name, and described as writing “on behalf of the GOT-IT clinical trial group”. The final trial report will include a GOT-IT contributor box, which will list all professionals that have contributed to the GOT-IT study for a minimum of one year.
For substudy papers, similar principles on authorship will apply.

15.2 PUBLICATION

The clinical study report will be used for publication and presentation at scientific meetings. Investigators have the right to publish orally or in writing the results of the study.

Summaries of results will also be made available to Investigators for dissemination within their clinics (where appropriate and according to their discretion).

15.3 PEER REVIEW

The protocol as undergone external peer review as part of the application process to the Health Technology Assessment Board for funding.

16 REFERENCES

1. NICE, *NICE clinical guideline 55: Intrapartum care: care of healthy women and their babies during childbirth.* 2012.
2. Cheung, W.M., et al., *The retained placenta: historical and geographical rate variations.* J Obstet Gynaecol, 2011. **31**(1): p. 37-42.
3. Stones, R.W., C.M. Paterson, and N.J. Saunders, *Risk factors for major obstetric haemorrhage.* Eur J Obstet Gynecol Reprod Biol, 1993. **48**(1): p. 15-8.
4. Combs, C.A., E.L. Murphy, and R.K. Laros, Jr., *Factors associated with postpartum hemorrhage with vaginal birth.* Obstet Gynecol, 1991. **77**(1): p. 69-76.
5. Lennox, C. and J. Marr, *Scottish Confidenital Audit of Severe Maternal Morbidity; reducing avoidable harm*, H.I. Scotland, Editor. 2012.
6. Abdel-Aleem, H., M.A. Abdel-Aleem, and O.M. Shaaban, *Tocolysis for management of retained placenta.* Cochrane Database Syst Rev, 2011(1): p. CD007708.
7. Group, I.C.S., *Research Priorities for Intrapartum Clinical Study Executive Group*. 2012.
8. Barnes, F., *Hour-Glass Contraction of the Uterus Treated with Nitrite of Amyl.* Br Med J, 1882. **1**(1107): p. 377.
9. Dufour, P., D. Vinatier, and F. Puech, *The use of intravenous nitroglycerin for cervico-uterine relaxation: a review of the literature.* Arch Gynecol Obstet, 1997. **261**(1): p. 1-7.
10. Chedraui, P.A. and D.F. Insuasti, *Intravenous nitroglycerin in the management of retained placenta.* Gynecol Obstet Invest, 2003. **56**(2): p. 61-4.
11. DeSimone, C.A., M.C. Norris, and B.L. Leighton, *Intravenous nitroglycerin aids manual extraction of a retained placenta.* Anesthesiology, 1990. **73**(4): p. 787.
12. Lowenwirt, I.P., R.M. Zauk, and S.M. Handwerker, *Safety of intravenous glyceryl trinitrate in management of retained placenta.* Aust N Z J Obstet Gynaecol, 1997. **37**(1): p. 20-4.
13. Peng, A.T., et al., *Intravenous nitroglycerin for uterine relaxation in the postpartum patient with retained placenta.* Anesthesiology, 1989. **71**(1): p. 172-3.
14. Rolbin, S.H., E.M. Hew, and A. Bernstein, *Uterine relaxation can be life saving.* Can J Anaesth, 1991. **38**(7): p. 939-40.
15. Bullarbo, M., J. Tjugum, and E. Ekerhovd, *Sublingual nitroglycerin for management of retained placenta.* Int J Gynaecol Obstet, 2005. **91**(3): p. 228-32.
16. Redick, L.F. and E. Livingston, *A new preparation of nitroglycerin for uterine relaxation.* Int J Obstet Anesth, 1995. **4**(1): p. 14-6.
17. Laslett, L.J. and L. Baker, *Sublingual nitroglycerin administered by spray versus tablet: comparative timing of hemodynamic effects.* Cardiology, 1990. **77**(4): p. 303-10.
18. Marmor, A., *Comparative evaluation of a new formulation of isosorbide dinitrate oral spray and sublingual nitroglycerin tablets.* Am J Cardiol, 1990. **65**(21): p. 43J-45J.
19. Curry, S.H., et al., *Plasma concentrations and hemodynamic effects of intravenous, sublingual, and aerosolized nitroglycerin in patients undergoing cardiac catheterization.* Biopharm Drug Dispos, 1993. **14**(2): p. 107-18.
20. Wight, L.J., et al., *A large scale comparative study in general practice with nitroglycerin spray and tablet formulations in elderly patients with angina pectoris.* Eur J Clin Pharmacol, 1992. **42**(3): p. 341-2.
21. Farley, A.E., C.H. Graham, and G.N. Smith, *Contractile properties of human placental anchoring villi.* Am J Physiol Regul Integr Comp Physiol, 2004. **287**(3): p. R680-5.
22. Bollapragada, S.S., et al., *Randomised placebo-controlled trial of outpatient (at home) cervical ripening with isosorbide mononitrate (IMN) prior to induction of labour--clinical trial with analyses of efficacy and acceptability. The IMOP study.* BJOG, 2009. **116**(9): p. 1185-95.
23. Glaser, B. and A. Strauss, *The Discovery of Grounded Theory*. 1967, Chicago: Aldine.
24. Hallowell, N., et al., *Distinguishing research from clinical care in cancer genetics: theoretical justifications and practical strategies.* Soc Sci Med, 2009. **68**(11): p. 2010-7.
25. Hallowell, N., et al., *Healthcare professionals' and researchers' understanding of cancer genetics activities: a qualitative interview study.* J Med Ethics, 2009. **35**(2): p. 113-9.

26. Strauss, A. and J. Corbin, *Basics of qualitative research: grounded theory procedures and techniques.* . 1990, London: Sage.
27. RCOG, *Obtaining valid consent to participate in research while in labour*, in *Clinical Governance Advice No.6a*. 2010, RCOG: London.
28. Vernon, G., Z. Alfirevic, and A. Weeks, *Issues of informed consent for intrapartum trials: a suggested consent pathway from the experience of the Release trial [ISRCTN13204258].* Trials, 2006. **7**: p. 13.
29. Weeks, A.D., et al., *Umbilical vein oxytocin for the treatment of retained placenta (Release Study): a double-blind, randomised controlled trial.* Lancet, 2010. **375**(9709): p. 141-7.

APPENDIX 1: Summary of Product Characteristics

SUMMARY OF PRODUCT CHARACTERISTICS

1 NAME OF THE MEDICINAL PRODUCT

Glyceryl Trinitrate Spray 400 micrograms/metered dose, sublingual spray.

2 QUALITATIVE AND QUANTITATIVE COMPOSITION

Active Ingredient

Glyceryl Trinitrate: 400 micrograms/metered dose

This product contains small amounts of ethanol (alcohol) less than 100mg per spray.

For full list of excipients, see Section 6.1

3 PHARMACEUTICAL FORM

Metered dose oromucosal (sublingual) spray solution

Small aerosol canister.

4 CLINICAL PARTICULARS

4.1 Therapeutic indications

Treatment of acute angina pectoris.

Prevention of inducible angina (e.g. physical effort, emotional stress, exposure to cold).

Route of administration

Oromucosal (Sublingual)

4.2 Posology and method of administration

Oromucosal dosage.

Before using Glyceryl Trinitrate Spray for the first time, the patient should check that the spray is working by pressing the pump button a few times until it produces a

fine mist of liquid. The patient should practice aiming the spray onto a tissue or similar item so that they will be able to aim it correctly under the tongue when they need to use it. If the patient does not need to use Glyceryl Trinitrate Spray very often, the spray should be checked regularly to see that it still works properly.

Adults including the Elderly

At the onset of an attack: one or two metered doses (400 to 800 micrograms glyceryl trinitrate) to be sprayed under the tongue for the relief of anginal pain while the breath is held. No more than three doses are recommended at any one time.

For the prevention of inducible angina (e.g. physical effort, emotional stress, exposure to cold) one or two 400 microgram metered doses sprayed under the tongue within 2-3 minutes of the event starting.

Children

Glyceryl Trinitrate Spray is not recommended for

children. Administration

During application the patient should rest, ideally in the sitting position. The canister should be held vertically with the valve head uppermost and the spray orifice as close to the mouth as possible. The dose should be sprayed under the tongue and the mouth should be closed immediately after each dose. The spray should not be inhaled. Patients should be instructed to familiarise themselves with the position of the spray orifice, which can be identified by the finger rest on top of the valve, in order to facilitate orientation for the administration at night.

4.3 Contraindications

Hypersensitivity to nitrates or to any of the excipients. Severe hypotension (systolic blood pressure lower than 90mm Hg) Hypotensive shock, severe anaemia, constrictive pericarditis, extreme bradycardia, Glucose-6-phosphate-Dehydrogenase-deficiency, cerebral haemorrhage and brain trauma, aortic and/or mitral stenosis and angina caused by hypertrophic obstructive cardiomyopathy. Circulatory collapse, cardiogenic shock and toxic pulmonary oedema.

Concomitant use with phosphodiesterase inhibtors such as Sildenafil, Tadalafil or Vardenafil.

4.4. Special Warnings and Precautions for Use

Tolerance to this drug and cross-tolerance to other nitrates may occur.

Glyceryl Trinitrate Spray should be administered with particular caution in:
- pericardial tamponade
- low filling pressures (e.g. acute myocardial infarction, left ventricular failure)

- tendency to dysregulation of orthostatic blood pressure
- diseases accompanied by an increase intracranial pressure (so far further pressure increase has been observed solely in high doses of glyceryl trinitrate).

  Alcohol should be avoided because of the hypotensive effect. Medical controls of the intraocular pressure of glaucoma-patients are advisable. Particular caution should also be exercised when using Glyceryl Trinitrate Spray in patients with volume depletion from diuretic therapy, severe hepatic or renal impairment and hypothyroidism.

4.5 Interaction with other medicinal products and other forms of interaction

Alcohol may potentiate the hypotensive effect. Vasodilators, antihypertensives, ß-blockers, calcium antagonists, neuroleptics tricyclic antidepressants and diuretics can increase nitrate induced hypotension.

The hypotensive effects of nitrates are potentiated by the concurrent administration of phosphodiesterase inhibitors, such as Sildenafil, Tadalafil or Vardenafil.

The bioavailability of dihydroergotamine may be increased by concomitant use of Glyceryl Trinitrate Spray, which can result in vasoconstriction since dihyroergotamine can antagonise the effects of glyceryl trinitrate. The concomitant administration of Glyceryl Trinitrate Spray and heparin can reduce the antithrombotic effect of heparin. Regular monitoring of coagulation parameters and adjustments of the heparin dose may be necessary.

In patients pretreated with organic nitrates a higher dose of glyceryl trinitrate may be necessary to achieve the desired haemodynamic effect.

4.6 Pregnancy and lactation

The safety of glyceryl trinitrate in human pregnancy, especially during the first trimester has not been established. It is not known whether glyceryl trinitrate is excreted into human breast milk. Glyceryl Trinitrate Spray should only be used after weighing the benefit for the mother against possible risks for the child. Nursing should be discontinued during treatment with this product.

4.7. Effects on Ability to Drive and Use Machines

The ability to react may be diminished because of the side effects or interactions due to the nitrates. This effect is potentiated by alcohol consumption. Therefore, driving and/or using machines should be avoided during treatment with Glyceryl Trinitrate Spray.

4.8 Undesirable effects

The following adverse reactions have been reported:

System Organ Class	Very Common (≥ 10%)	Common (≥ 1% <10%)	Uncommon (≥ 0.1% <1%)	Rare (≥0.01% <0.1%)	Very Rare (≥0.01% <0.01%)
Nervous System Disorders	Headache	Vertigo			
Skin and Subcutaneous Tissue Disorders		Facial Flushing			
Vascular Disorders		Dizziness		Postural Hypotension	
General Disorders and Administration Site Conditions		Weakness	Burning Sensation Stinging Sensation Tongue Blistering		
Gastrointestinal Disorders		Nausea			
Cardiac Disorders				Tachycardia Bradycardia	
Skin and Subcutaneous Tissue Disorders				Allergic Skin Reactions	Exfoliative Dermatitis

Rarely collapse states with bradycardia and syncope, a severe fall in blood pressure accompanied by an enhancement of the anginal symptoms may occur.

Use of Glyceryl Trinitrate Spray may give rise to transient hypoxaemia and, in patients with coronary heart disease, ischaemia as a result of a relative redistribution of the bloodstream, which is to hypoventilated alveolar areas.

Tolerance development and the occurrence of crossed tolerance of other nitro compounds have been found in chronic, continuous treatment using high doses. To avoid a decrease in efficacy or a loss of efficacy, high continuous doses should be avoided.

Reporting of suspected adverse reactions
Reporting suspected adverse reactions after authorisation of the medicinal product is important. It allows continued monitoring of the benefit/risk balance of the medicinal product. Healthcare professionals are asked to report any suspected adverse reactions via the Yellow Card Scheme at: www.mhra.gov.uk/yellowcard.

4.9 Overdose

Signs and Symptoms
Flushing, severe headache, vertigo, tachycardia, a feeling of suffocation, hypotension, fainting and rarely cyanosis and methaemoglobinaemia may occur. In a few patients, there may be a reaction comparable to shock with nausea, vomiting, weakness, sweating and syncope.

Treatment
Recovery often occurs without special treatment. Hypotension may be corrected by elevation of the legs to promote venous return. Methaemoglobinaemia should be treated by intravenous methylthioninium chloride and/or toluidine blue. Symptomatic treatment should be given for respiratory and circulatory defects in more serious cases.

5 PHARMACOLOGICAL PROPERTIES

5.1 Pharmacodynamic properties

ATC-Code: C0IDA02

Glyceryl trinitrate acts on vascular smooth muscles to produce arterial and venous vasodilation. The vasodilation results in a reduction of venous return and an improvement in myocardial perfusion with the result of a reduction in the work performed by the heart and hence reduced oxygen demand.

5.2. Pharmacokinetic Properties

Glyceryl trinitrate is rapidly absorbed through the buccal and sublingual mucosa, and in man peak concentrations in plasma are observed within four minutes of sublingual administration.

The absolute bioavailability after sublingual administration is approximately 39%. After sublingual administration the plasma levels have shown a wide range of intra and inter-individual variability. The compound is extensively metabolised by liver enzymes and has a plasma half life of 1-3 minutes. The principle mechanism of metabolism involves denitration.

5.3 Preclinical safety data

Preclinical date reveal no special hazard for human based conventional studies of safety pharmacology, repeated dose toxicity, genotoxicity, carcinogenic potential, or toxicity to reproduction.

6. PHARMACEUTICAL PARTICULARS

6.1. List of Excipients

Peppermint oil
Propellant HFC 134A (1,1,1.2 Tetrafluoroethane)
Ethanol

6.2. Incompatibilities

None known.

6.3 Shelf-Life

Three years

6.4 Special precautions for storage

Do not store above 25°C. Do not refrigerate or freeze.

6.5 Nature and contents of container

Internally lacquered monobloc Aluminium pressurised container sealed with a metered spray valve.

The product is presented in packs with one metered dose spray.

One metered dose spray (= one aluminium container) contains either 1760.0mg of solution (according to 11400.0mg of solution and propellant) providing 200 single metered doses, or 1584.0mg of solution (according to 10260.0mg of solution and propellant) providing 180 metered doses.

6.6 Special precautions for disposal

Glyceryl Trinitrate is an aerosol spray and contains a pressurised liquid. Do not expose to temperatures higher than 50 °C, and do not pierce the canister, even when empty. It should not be sprayed at a naked flame or any incandescent material. Patients, especially those who smoke should be warned not to use glyceryl trinitrate spray near a naked flame.

7 MARKETING AUTHORISATION HOLDER

Ayrton Saunders Ltd
9 Arkwright Road
Astmoor Industrial Estate
Runcorn
Cheshire
WA7 1NU

8. MARKETING AUTHORISATION NUMBER(S)

PL 16431/0018

9 Date of the first authorisation or renewal

15/09/2005

10 DATE OF REVISION OF THE TEXT

27/08/2015
