# Supplementary material for: Nitroglycerin for treatment of retained placenta: A randomised, placebo-controlled, multicentre, double-blind trial in the UK
Source: PLoS Med. 2019 Dec 30;16(12):e1003001. doi: 10.1371/journal.pmed.1003001 (PMC6936786; doi:10.1371/journal.pmed.1003001)
Supplement: S2 Text — (PDF) [file pmed.1003001.s011.pdf]

S2_Text
GOT-IT statistical analysis plan

Centre for Healthcare Randomised Trials

Got-it

A pragmatic group sequential placebo controlled randomised trial to determine the effectiveness of Glyceryl trinitrate for retained placenta.

GOT-IT TRIAL
Glycerine trinitrate fOr reTaIned placenTa

# Statistical Analysis Plan

SAP Version: 1.0
Protocol Version: 8.0
Protocol Date: 27th April 2016

| Approved by: | | |
|---|---|---|
| Dr Fiona Denison<br>Chief Investigator | (signed) | 09/Nov/2017<br>date |
| Professor John Norrie<br>Individual Responsible for Statistical Review | (signed) | 09 NOV 2017<br>date |
| Professor Graeme MacLennan<br>CHaRT director | (signed) | [illegible] November 2017<br>date |

## Contents


**1 Study Objectives** 2

**2 Study Methods** 2
- 2.1 General Study Design . . . 2
- 2.2 Group Sequential Design . . . 2
- 2.3 Randomisation and Blinding . . . 3

**3 Outcome Measures** 3
- 3.1 Primary outcome . . . 3
- 3.2 Secondary outcomes . . . 4
- 3.3 Timing of outcome measurements . . . 4
- 3.4 Adverse events . . . 4

**4 Sample Size and Power Calculations** 5

**5 Statistical Methods** 5
- 5.1 Primary outcomes . . . 6
- 5.2 Secondary outcomes . . . 6
- 5.3 Subgroup analyses . . . 6
- 5.4 Missing Data . . . 6

**6 Dummy Tables** 7


## List of Tables


1 Recruitment by centre - n(%) . . . 7

2 Baseline characteristics . . . 8

3 Primary clinical outcome of need for MROP at 15 minutes post administration of study drug . . . 8

4 Primary safety outcome of blood loss between administration of treatment and transfer to postnatal ward or other clinical area . . . 8

5 Primary patient-sided outcome of satisfaction with treatment before discharge and at 6 weeks . . . 8

6 Primary patient-sided outcome of side effect profile before discharge and at 6 weeks . 9

7 Secondary clinical outcomes . . . 9


## List of Figures


1 Group sequential design . . . 3

# 1 Study Objectives

The overall aim of this study is to determine the clinical effectiveness and cost effectiveness of sublingual Glyceryl Trinitrate (GTN) spray compared with placebo in reducing the need for manual removal of placenta (MROP) in women with a retained placenta (RP) after vaginal delivery following failure of current management (defined as a third stage of labour lasting more than 30 minutes after active management or 60 minutes after physiological followed by active management respectively).

# 2 Study Methods

## 2.1 General Study Design

There will be an internal pilot RCT, full details of which are in the protocol. The purpose of the pilot is to provide reassurances about all of the trial processes and the results will be reported to the Data Monitoring Committee (DMC), on whose recommendation the trial will be expanded to the full study design.

GOT-IT is a randomised, placebo controlled, double-blind pragmatic trial. Due to the sparse nature of poor quality data for the use of GTN for RP, treatment and control effect rate, there is at present considerable uncertainty in designing a definite trial. We therefore believe that a group sequential design is appropriate given the lack of evidence currently available. We believe that this is the ideal trial design because it enables us to present the maximum size of trial that is needed, alongside a flexible group sequential approach that allows the trial to terminate early for one of two scenarios. The first scenario is overwhelming evidence of benefit (due perhaps to a large treatment effect and/or less variability in the outcome measure). The second scenario is due to a suitably defined futility - that is, having got a certain way to the maximum trial size, we are confident that a large treatment effect is implausible, and that the current estimate of the treatment effect is sufficiently precise to be convincing, allowing the trial to terminate early.

## 2.2 Group Sequential Design

Following detailed discussions with the DMC, we will use a Lan-DeMets alpha spending approach [1] with OBrien Fleming boundaries [2], as a two-sided test, with efficacy and futility boundaries. There will be 5 interim evaluations, equally spaced at 215, 429, 644, 858 and 1073 participants. With the O'Brien Fleming boundaries, there is the possibility of stopping early at the 215 and 429 looks for example, but only for very strong evidence (either way) and if there is still the need to go to full trial size the ability to determine a treatment effect near the 5% level of significance by 'wasting the alpha' in pointless early evaluations will not be compromised.

The general form of the group sequential design is; participants are randomised between two treatment groups, experimental (GTN spray) and a comparator (placebo). At the $i^{th}$ interim analysis, when the required number of participant responses have been observed the test statistic is calculated. If this statistic crosses the pre-specified boundaries, the trial can be stopped either to reject the null hypothesis or fail to reject the null hypothesis. For this study, it is possible to stop for efficacy at all of the interim evaluations, however only at the $3^{rd}$ and last for futility. Figure 1 summarises the design.

Figure 1. Group sequential design

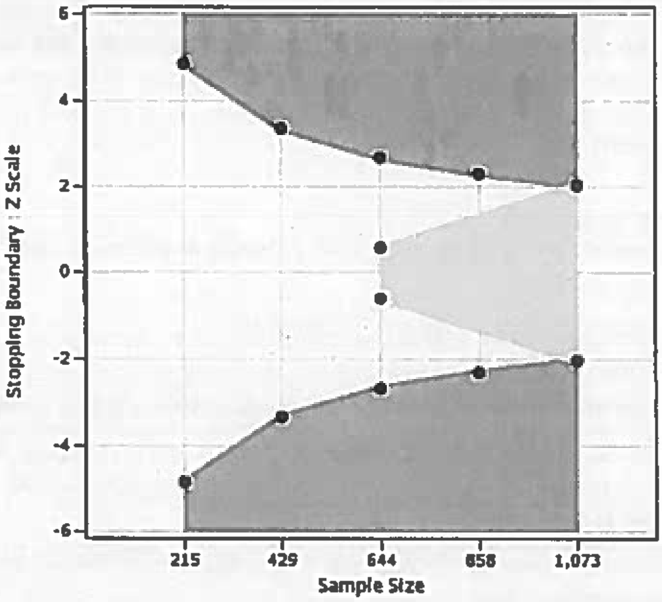


## 2.3 Randomisation and Blinding

Randomisation packs will be ordered from pharmacy and kept on the labour ward. Study drugs will be provided to site pharmacies in pre-packed randomised, permuted blocks. Once a participant is recruited the study drug will be allocated by taking the next available treatment pack from the shelf. The study will be performed double masked so neither the patient nor the investigator will know which treatment has been allocated. Breaking of the study masking will only be performed where knowledge of the treatment is absolutely necessary for safe management of the patient.

Central unblinding procedures will be maintained by the Centre for Healthcare Randomised Trials (CHaRT) who will hold the randomisation list for the trial. Unblinding (emergency or otherwise) can be carried out by a senior clinician (normally a consultant). The senior clinician will call an Interactive Voice Response System (IVRS) and enter their name, the reason for unblinding, and the study drug number into the automated system. This phone line will be available at all times. The unblinding will be recorded on the trial database and the trial manager will be informed. Unless there is a clinical requirement, the masking will not be broken until after data entry is complete, the validity of the data is checked, all queries resolved and the patient populations agreed.

# 3 Outcome Measures

## 3.1 Primary outcome

- **Clinical:** need for MROP, defined as the placenta remaining undelivered 15 minutes post study treatment and/or being required within 15 minutes of treatment due to safety concerns.

- **Safety:** measured blood loss between administration of treatment and transfer to the post-natal ward or other clinical area (e.g. labour ward high dependency)
- **Patient-centred:** satisfaction with treatment and side effect profile assessed by questionnaire. It will be based on qualitative studies and previously used questionnaires to assess satisfaction [3] .

## 3.2 Secondary outcomes

- **Clinical:**

    i i. Fall in haemoglobin of more than 15% between recruitment and the first postnatal day

    ii Time from randomisation to delivery of placenta

    iii MROP in theatre

    iv Need for earlier than planned MROP on the basis of the clinical condition

    v Fall in systolic or diastolic blood pressure of more than 15mmHg and/or increase in pulse of more than 20 beats/minute between baseline and 5 and 15 minutes post-administration of active/placebo treatment.

    vi Need for blood transfusion between time of delivery and discharge from hospital

    vii Need for general anaesthesia

    viii Maternal pyrexia (one or more temperature readings of more than 38C within 72 hrs of delivery or discharge from hospital if discharge occurs sooner)

    ix Sustained uterine relaxation after removal of placenta using uterotonics.

## 3.3 Timing of outcome measurements

| | Baseline | Randomisation | 5 minutes* | 15 minutes* | Before transfer | Before discharge | 6 weeks |
|---|---|---|---|---|---|---|---|
| BP, pulse, temperature | ✓ | | ✓ | ✓ | | | |
| Full blood count | ✓ | | | | | ✓ | |
| Treatment allocated & administered | | ✓ | | | | | |
| Blood loss | | | | | ✓ | | |
| Need for MROP | | | | ✓ | | | |
| Patient rated side-effects | | | | | | ✓ | ✓ |
| Patient rated satisfaction | | | | | | ✓ | ✓ |
| Adverse events | | | | | | ✓ | ✓ |

* post administration of study drug

## 3.4 Adverse events

An adverse event (AE) is any untoward medical occurrence in a clinical trial participant which does not necessarily have a causal relationship with an investigational medicinal product (IMP).

An **adverse reaction** (AR) is any untoward and unintended response to an IMP which is related to any dose administered to that participant.

A **serious adverse event** (SAE), **serious adverse reaction** (SAR) is any AE or AR that at any dose:

- results in death of the clinical trial participant;
- is life threatening*;
- requires in-patient hospitalisation† or prolongation of existing hospitalisation;
- results in persistent or significant disability or incapacity;
- consists of a congenital anomaly or birth defect;
- results in any other significant medical event not meeting the criteria above.

* Life-threatening in the definition of an SAE or SAR refers to an event where the participant was at risk of death at the time of the event. It does not refer to an event which hypothetically might have caused death if it were more severe.
† Any hospitalisation that was planned prior to randomisation will not meet SAE criteria. Any hospitalisation that is planned post-randomisation will meet the SAE criteria.

Participants will be asked about the occurrence of AE/SAEs prior to discharge from the hospital and in the 6 week postnatal questionnaire. These will be summarised and presented.

## 4 Sample Size and Power Calculations

We believe that a 10% decrease of women needing manual removal of the placenta will be sufficient advantage to change clinical practice and result in the widespread uptake of this simple, safe and cheap drug intervention (2 puffs (800g) sublingual GTN spray administered within minutes of a diagnosis of suspected retained placenta). From a statistical perspective, the maximum variability in a binary outcome (need for surgical intervention yes or no) occurs at a 50% rate in the placebo spray arm. On a fixed sample approach at 90% power and 5% level of significance, this would need 1038 women (519 in each group) to demonstrate a 10% change from 50% on placebo to 40% on GTN spray. Since the outcome is recorded within minutes of the intervention on the hospital systems (surgery took place yes/no), we anticipate minimal (if any) loss to follow up. So we can be confident that there is no need for a trial larger than this, except for the need for a small uplift to a possible maximum of 1073 women to allow for the multiple sequential evaluations of the data, to reliably answer this question.

## 5 Statistical Methods

The statistical analysis will be based on all participants as randomised, irrespective of subsequent compliance with the treatment allocated (i.e. following the intention-to-treat principle). Therefore all participants will remain in their allocated group for analysis.

Statistical significance will be at the 5% level with corresponding 95% confidence intervals (CI) derived.

Treatment groups will be described at baseline and follow-up using means (with standard deviations), medians (with inter-quartile ranges) and numbers (with percentages) where appropriate.

The interim analyses for the DMC will be specified within their DMC Charter, and results of these interim analyses will be in strict confidence (no member of the research team apart from the study statistician will be aware of the contents of these analyses). Advice on the final sample size will be revealed to the Trial Steering Committee (TSC).

## 5.1 Primary outcomes

The primary clinical outcome of need for MROP will be analysed using logistic regression, and odds ratios of the treatment effect, together with 95% CIs and associated likelihood ratio P-value, will be estimated. Other primary outcomes will be analysed using generalised linear models appropriate to the distribution of each outcome and including centre as a random effect. These models will adjust for relevant baseline factors.

## 5.2 Secondary outcomes

Secondary outcomes will be analysed using the appropriate generalised linear model as for the primary outcomes, using 5% significance level, and corresponding 95% CIs will be derived.

## 5.3 Subgroup analyses

Planned subgroup analyses are intended to explore potential effect modifications of previous C-section and gestation at delivery ($<36$ and $\geq 36$ weeks gestation). A stricter level of statistical significance will be applied (1% with 99% CI derived).

## 5.4 Missing Data

Sensitivity analyses will assess the robustness of the findings to any missing data. However for the primary clinical outcome this is expected to be virtually nil, as the outcome is measured within minutes of taking the intervention.

## 6 Dummy Tables

Table 1. Recruitment by centre - n(%)

| Centre | GTN spray | Placebo |
|---|---|---|
| Edinburgh | | |
| Glasgow | | |
| Manchester | | |
| . | | |
| . | | |
| . | | |
| . | | |

Table 2. Baseline characteristics

| | GTN spray N= | Placebo N= |
|---|---|---|
| Age (years) - mean(SD) | | |
| BMI - mean(SD) | | |
| Smoker - n(%) | | |
| Current | | |
| Ex-smoker | | |
| Never | | |
| Alcohol use in pregnancy - n(%) | | |
| Yes | | |
| No | | |
| Ethnicity - n(%) | | |
| White | | |
| Asian | | |
| Black | | |
| Mixed | | |
| Chinese | | |
| Other | | |
| Blood Pressure (mmHg) - mean(SD) | | |
| Systolic | | |
| Diastolic | | |
| Pulse (bpm) - mean(SD) | | |
| Temperature (C) - mean(SD) | | |
| Haemoglobin (g/dL) ) - mean(SD) | | |
| Previous pregnancy - n(%) | | |
| Previous retained placenta - n(%) | | |
| Previous placenta praevia/ accrete - n(%) | | |

Table 3. Primary clinical outcome of need for MROP at 15 minutes post administration of study drug

| | GTN spray N = | Placebo N = | Odds ratio | 95% CI | p-value |
|---|---|---|---|---|---|
| Proportion of women needing MROP –n(%)[1] | | | | | |

[1] Defined as proportion of women needing MROP because the placenta remains undelivered 15 minutes post study treatment and/or is required within 15 minutes of treatment due to safety concerns.

Table 4. Primary safety outcome of blood loss between administration of treatment and transfer to postnatal ward or other clinical area

| | GTN spray N = | Placebo N = | Odds ratio | 95% CI | p-value |
|---|---|---|---|---|---|
| Blood loss (ml) –n(%) | | | | | |
| < 500 | | | | | |
| 500 - 1000 | | | | | |
| > 1000 | | | | | |

Table 5. Primary patient-sided outcome of satisfaction with treatment before discharge and at 6 weeks

| | GTN spray N= | Placebo N= | Odds ratio | 95% CI | p-value |
|---|---|---|---|---|---|
| Would you recommend this treatment (study drug) to a friend/relative? –n(%) | | | | | |
| Discharge | | | | | |
| 6 weeks | | | | | |

Table 6. Primary patient-sided outcome of side effect profile before discharge and at 6 weeks

| | GTN spray N= | Placebo N= | Odds ratio | 95% CI | p-value |
|---|---|---|---|---|---|
| Feeling sick –n(%) | | | | | |
| Discharge | | | | | |
| 6 weeks | | | | | |
| Palpitations/heart racing –n(%) | | | | | |
| Discharge | | | | | |
| 6 weeks | | | | | |

Table 7. Secondary clinical outcomes

| | GTN spray N= | Placebo N= | Estimate | 95% CI | p-value |
|---|---|---|---|---|---|
| More than 15% fall in haemoglobin | | | | | |
| Time from randomisation to delivery of placenta (mins) | | | | | |
| MROP in theatre | | | | | |
| Need for earlier than planned MROP | | | | | |
| Fall in systolic, diastolic, or pulse[1] | | | | | |
| Blood transfusion | | | | | |
| General anaesthesia | | | | | |
| Maternal pyrexia | | | | | |
| Sustained uterine relaxation | | | | | |

Values are n(%) and estimate are odds ratio for dichotomous variables or mean(SD) and mean differences for continuous.

[1] Fall in systolic or diastolic blood pressure of more than 15mmHg and/or increase in pulse of more than 20 beats/minute between baseline and 5 and 15 minutes post-administration of active/placebo treatment

## References


[1] K. K. G. LAN and D. L. DeMets. Discrete sequential boundaries for clinical trials. *Biometrika*, 70(3):659–663, 1983. doi: 10.1093/biomet/70.3.659.

[2] Peter C. O'Brien and Thomas R. Fleming. A multiple testing procedure for clinical trials. *Biometrics*, 35(3):549–556, 1979.

[3] SS Bollapragada, F MacKenzie, JD Norrie, O Eddama, S Petrou, M Reid, and JE Norman. Randomised placebo-controlled trial of outpatient (at home) cervical ripening with isosorbide

mononitrate (IMN) prior to induction of labour – clinical trial with analyses of efficacy and acceptability. the IMOP study. *BJOG: An International Journal of Obstetrics and Gynaecology*, 116(9):1185–115, 2009. doi: 10.1111/j.1471-0528.2009.02216.x.
