# Supplementary material for: Nitroglycerin for treatment of retained placenta: A randomised, placebo-controlled, multicentre, double-blind trial in the UK
Source: PLoS Med. 2019 Dec 30;16(12):e1003001. doi: 10.1371/journal.pmed.1003001 (PMC6936786; doi:10.1371/journal.pmed.1003001)
Supplement: S3 Text — (DOCX) [file pmed.1003001.s012.docx]

**S3 Text:**

**Trial Oversight Committees: Trial Steering Committee and Data Monitoring Committee**

The GOT-IT investigators are very grateful to the following for their participation in the independent Trial Steering and independent Data Monitoring Committees.

**Trial Steering Committee**

Chair - Professor Andrew Shennan, King’s College London

Dr Kitty Bloemenkamp, University of Leiden

Dr Claire Snowdon, London School of Hygiene and Tropical Medicine

Professor Debra Bick, King’s College London

Dr Louise Brown, Population Health Sciences, University College London

**Data Monitoring Committee**

Chair – Professor Phillip Bennett

Professor Lucy Chappell, King’s College London

Professor Amanda Farrin, University of Leeds
