# Supplementary material for: Nitroglycerin for treatment of retained placenta: A randomised, placebo-controlled, multicentre, double-blind trial in the UK
Source: PLoS Med. 2019 Dec 30;16(12):e1003001. doi: 10.1371/journal.pmed.1003001 (PMC6936786; doi:10.1371/journal.pmed.1003001)
Supplement: S4 Text — (DOCX) [file pmed.1003001.s013.docx]

S4 Text:

GOT-IT Trial Discharge Questionnaire


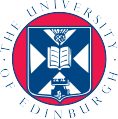
THE UNIVERSITY

*of* EDINBURGH

Got-it

| PIN |  |  |  |  |  |
| --- | --- | --- | --- | --- | --- |

**GOT-IT Trial Discharge Questionnaire**

Thank you for taking part in the GOT-IT Trial.

This questionnaire should only take you about 5 minutes to complete and it will ask you about:

1. Your experience of labour.
2. Your experience of the treatment you received for retained placenta.

# How to fill in this questionnaire:

- Please read each question carefully and fill in the answer which best describes how you feel.
- Please try to answer all of the questions.
- There are no right or wrong answers. If you are unsure about how to answer a question please put the best answer you can.
- If you make a mistake or change your mind about an answer, shade out the answer completely and mark the correct box/write the new answer alongside it.

# Completing and returning the questionnaire:

We would be very grateful if you could complete and return the questionnaire to us as soon as you can. Please fill in the date you complete this questionnaire:

|  | |  | |  | | | |
| --- | --- | --- | --- | --- | --- | --- | --- |
| D | D | M | M | Y | Y | Y | Y |

If you have any questions, then please do contact us on:

Dr Fiona Denison (Chief Investigator) or Kathryn Carruthers (GOT-IT Trial Manager)

Tel: 0131 242 6449; Tel: 0131 242 6753;

Thank you once again for helping with our research. The GOT-IT Trial Team

*Please continue overleaf.*

| PIN |  |  |  |  |  |
| --- | --- | --- | --- | --- | --- |

# The Birth of your Baby

Thinking back to your labour and the birth of your baby:

1. How do you think your birth went? Please circle the number which best describes your experience:

**1 2 3 4 5**

**Very Easy Easy Neither Easy Difficult Very Difficult**

**nor difficult**

1. How satisfied were you with your birth experience overall? Please circle the number which best describes your experience:

**1 2 3 4 5**

**Very Satisfied Satisfied Neither Satisfied Dissatisfied Very Dissatisfied**

**nor dissatisfied**

1. Did you have your placenta removed by an operation in theatre?

Yes No

# Your Treatment for Retained (stuck) Placenta

When you took part in the study, you were given a treatment (study drug) to spray under your tongue to help your placenta to come out.

We would like you to find out whether you experienced any symptoms after taking the study drug and before you had any operation.

We would like you to circle Yes, No or Can’t remember for each of the symptoms below. Did you experience?

| I. | Headache | Yes | No | Can’t remember |
| --- | --- | --- | --- | --- |
| II. | Feeling sick | Yes | No | Can’t remember |
| III. | Hot and bothered | Yes | No | Can’t remember |
| IV. | Dizziness | Yes | No | Can’t remember |
| V. | Sleepiness | Yes | No | Can’t remember |
| VI. | Palpitations/heart racing | Yes | No | Can’t remember |
| VII. | Other |  |  |  |

Please specify:

Would you recommend this treatment (study drug) to a friend/relative? Yes No

If you wish to provide further information, please do so in the box below:
