# Supplementary material for: Nitroglycerin for treatment of retained placenta: A randomised, placebo-controlled, multicentre, double-blind trial in the UK
Source: PLoS Med. 2019 Dec 30;16(12):e1003001. doi: 10.1371/journal.pmed.1003001 (PMC6936786; doi:10.1371/journal.pmed.1003001)
Supplement: S5 Text — (DOCX) [file pmed.1003001.s014.docx]

S5 Text

GOT-IT Trial Postnatal Questionnaire


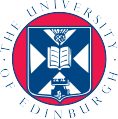
THE UNIVERSITY

*of* EDINBURGH

Got-it

| PIN |  |  |  |  |  |
| --- | --- | --- | --- | --- | --- |

**GOT-IT Trial Postnatal Questionnaire**

Thank you for taking part in the GOT-IT Trial.

You might remember that we said we would send you a questionnaire as part of the research. This questionnaire should only take you about 15 minutes to complete and it will ask you about:

1. Your experience of labour.
2. Your experience of the treatment you received for retained placenta.
3. Your health since you took part in the trial.

# How to fill in this questionnaire:

- Please read each question carefully and fill in the answer which best describes how you feel.
- Please try to answer all of the questions.
- There are no right or wrong answers. If you are unsure about how to answer a question please put the best answer you can.
- If you make a mistake or change your mind about an answer, shade out the answer completely and mark the correct box/write the new answer alongside it.

# Completing and returning the questionnaire:

We would be very grateful if you could complete and return the questionnaire to us as soon as you can.

## Please fill in the date you complete this questionnaire:

|  | |  | |  | | | |
| --- | --- | --- | --- | --- | --- | --- | --- |
| D | D | M | M | Y | Y | Y | Y |

There is a stamped addressed envelope included so that you can return it to us. If you have any questions, then please do contact us on:

Dr Fiona Denison (Chief Investigator) or Kathryn Carruthers (GOT-IT Trial Manager)

Tel: 0131 242 6449; Tel: 0131 242 6753;

Thank you once again for helping with our research. The GOT-IT Trial Team

*Please continue overleaf.*

| PIN |  |  |  |  |
| --- | --- | --- | --- | --- |

# The Birth of your Baby

Thinking back to your labour and the birth of your baby

1. How do you think your birth went? Please circle the number which best describes your experience:

**1 2 3 4 5**

**Very Easy Easy Neither Easy Difficult Very Difficult**

**nor Difficult**

1. How satisfied were you with your birth experience overall? Please circle the number which best describes your experience:

**1 2 3 4 5**

**Very Satisfied Satisfied Neither Satisfied Dissatisfied Very Dissatisfied**

**nor Dissatisfied**

1. Did you have your placenta removed by an operation in theatre?

Yes No

# Your Treatment for Retained (stuck) Placenta

When you took part in the study, you were given a treatment (study drug) to spray under your tongue to help your placenta to come out.

## We would like you to find out whether you experienced any symptoms after taking the study drug and

***before* you had any operation.**

We would like you to circle Yes, No or Can’t remember for each of the symptoms below.

## Did you experience?

| I. | Headache | Yes | No | Can’t remember |
| --- | --- | --- | --- | --- |
| II. | Feeling sick | Yes | No | Can’t remember |
| III. | Hot and bothered | Yes | No | Can’t remember |
| IV. | Dizziness | Yes | No | Can’t remember |
| V. | Sleepiness | Yes | No | Can’t remember |
| VI. | Palpitations/heart racing | Yes | No | Can’t remember |
| VII. | Other |  |  |  |

Please specify:

Would you recommend this treatment (study drug) to a friend/relative? Yes No

| PIN |  |  |  |  |
| --- | --- | --- | --- | --- |

# Health

We would like to know about your health service use and any treatment that you are having, or have had,

**for your own health** since you were discharged from hospital following the birth of your baby.

## Since you returned home following the birth of your baby:

I. Have you seen or contacted **a midwife or health visitor** because of **your own health**? **(please do not count routine visits)** Yes No

If ‘Yes’, please give details below:

How many times did you call a midwife?

How many times did you call a health visitor?

How many times did a midwife come out to see you?

How many times did a health visitor come out to see you?

I. Have you seen or contacted **a GP** because of **your own health** since you returned home? **(please do not count routine visits)** Yes No

If ‘Yes’, please give details below:

How many appointments did you attend with a GP?

How many times did a GP visit you at home?

How many times did you have a telephone conversation with a GP?

1. Have you been prescribed any of the following types of medication as a result of any infection, bleeding or pain you have experienced?

| **Type of** | **Have you been** | **Name of** | **Quantity** |
| --- | --- | --- | --- |
| **medication** | **prescribed this?** | **medication** | **prescribed** |
| Antibiotics | Yes No |  |  |
| Pain killers | Yes No |  |  |

1. Have you had any **hospital outpatient appointments** because of **your own health** since you returned home? (please do not count routine appointments for pre-existing health conditions)

Yes No

If ‘Yes’, how many outpatient appointments have you had?

1. Have you been **re-admitted to hospital** because of **your own health** since you returned home? Yes No

If ‘Yes’, please give details overleaf:

| PIN |  |  |  |  |
| --- | --- | --- | --- | --- |

| *3. Health (continued)* |  | |
| --- | --- | --- |
| **Hospital and ward (if known)** | **Reason you were admitted to hospital** | **Number of nights** |

1. If you wish to provide further information, please do so below:
