# Supplementary material for: Nitroglycerin for treatment of retained placenta: A randomised, placebo-controlled, multicentre, double-blind trial in the UK
Source: PLoS Med. 2019 Dec 30;16(12):e1003001. doi: 10.1371/journal.pmed.1003001 (PMC6936786; doi:10.1371/journal.pmed.1003001)
Supplement: Attachment — Submitted filename: GOT_IT_rebuttal_FINAL.docx [file pmed.1003001.s015.docx]

Requests from the editors:

*1. The following outcomes measures pertaining to the economic outcomes appear to differ between the submitted manuscript and the trial registry. Please clarify and explain the discrepancy. If the outcomes were not prespecified in the protocol, please indicate that they were post hoc and explain why they were added. Post hoc comparisons should be presented as hypothesis generating rather than conclusive.  

Specifically, a primary economic outcome of comparing GTN vs standard practice in terms of net incremental cost to UK NHS (page 7, Lines 174-176).  However, the following primary and secondary outcomes included in the trial registry are slightly different ([http://www.isrctn.com/ISRCTN88609453](http://www.isrctn.com/ISRCTN88609453)): Primary outcome: 4. Economic: net incremental costs (or cost savings) to the National Health Service of using GTN versus standard practice. Costs will include GTN (dose and time to administer drug, monitor woman and deliver the placenta if effective), MROP, and further health service resource use as measured by the 6-week postnatal questionnaire; Secondary outcome: 2. Costs: the mean costs will be summarised by treatment allocation group, and the incremental cost (cost saving) associated with the use of GTN will be estimated using an appropriately specified general linear model. The cost data will be presented alongside the primary and secondary outcome data in a cost-consequence
balance sheet, indicating which strategy each outcome favours (6-week postnatal questionnaire).*  

R1: Further to our recent email correspondence with the handling Editor, and discussed within team, we clarify the economic analyses presented below. We are keen to retain these within our manuscript, but would be prepared to remove if requested by the Editor.

The intended primary economic outcome has been presented in the manuscript, which is the expected incremental cost to the NHS of adopting GTN, net of any downstream costs or costs savings arising from its use. This is what is presented in the final row of Table 3. The preceding rows summarise the major cost categories by treatment allocation. With respect to the methods for estimating the cost difference (the net increment), whilst a generalised linear model was initially proposed to deal with potentially heavily skewed cost data, this was not found to be the case. Further, with a relatively large sample size, ordinary least squares with robust standard errors is expected to give consistent estimates. This was confirmed by generalised linear models with gamma family distribution and/or log link functions giving very similar estimates to the ordinary least squares. For this reason, we utilised this the ordinary linear regression analysis approach. Clarification has been added to the section of the manuscript summarising the economic analysis.

With the respect to the original plans to present costs in relation to the other primary and secondary outcomes in a summary cost-consequence balance sheet, this was in anticipation of significant trade-offs between the expected costs and the various clinical and safety outcomes. In light of the negative clinical findings and potential safety concern for GTN, we do not believe this would add anything to the paper. However, it can be provided as a supplementary table if the editors wish.

*C2. Title: Please make it clear in the title that the trial took place in the UK.*

R2: The title has been amended to:

Glyceryl trinitrate for treatment of retained placenta: a randomized, placebo-controlled, multicentre double blind trial in the UK

*C3. Abstract Line 44: October 14 should be October 2014.*

R3: The abstract has been amended to October 2014 (line 46).

*C4. Abstract Line 43-44: The second instance of “was undertaken” is a typo- please correct.  Also, “29 delivery units” should read “at 29 delivery units”.*

R4: The abstract has been amended to at 29 delivery units in the UK.

*C5. Abstract Results: Please quantify the main results (with 95% CIs and p values) for the adverse outcomes presented (e.g. palpitations, adverse events).*

R5: The 95% CI and p values are already presented for palpitations.

However, as requested, we have added in the 95% CI and p values for the adverse events. This is now detailed in the statistical analysis as a post-hoc analysis. Note that the P-values should be interpreted descriptively, as we did not have a hypothesis to test about each adverse event, and care should be taken with not over interpreting these data due to the substantial multiple comparisons issue. We can add this caveat in the text if the Editors agree.

*C6. Abstract methods and results: Please indicate the outcomes assessed at follow up, and length and method of follow up.*

R6: As requested, we have clarified the outcomes assessed at follow-up and length and method of follow-up (lines 52-59).

*C7. Abstract methods and results: Please indicate the locations (i.e. in which cities) of the maternity wards involved in the study.*

C7: As requested, we have added the locations (i.e. in which cities) of the maternity wards involved in the study (lines 46-50). We have added further detail in a new supplemental table 2.

*C8. At this stage, we ask that you include a short, non-technical Author Summary of your research to make findings accessible to a wide audience that includes both scientists and non-scientists. The Author Summary should immediately follow the Abstract in your revised manuscript.*

R8

**Why was this study done?**

- A retained placenta can cause clinically important bleeding in women following a vaginal birth
- The only effective treatment for a retained placenta is for it to be removed by an operation.
- In many parts of the world, surgery is not possible meaning that women die from this condition

**What did the researchers do and find?**

- We undertook a trial to assess whether a drug (glyceryl trinitrate) to relax the womb would be an effective, safe and acceptable medical treatment for retained placenta and would avoid the need for surgical removal.
- We recruited 1107 women with retained placenta and randomised them to treatment with sublingual glyceryl trinitrate or placebo spray to treat retained placenta.
- We found that glyceryl trinitrate was not effective as a medical treatment for retained placenta

**What do these findings mean?**

- The majority of women with a retained placenta following vaginal delivery need to have it removed by an operation.
- There remains a need for a new, safe and effective medical treatment for retained placenta for those women who live in settings where operative treatment if not available.

*C9. Methods and Results: Please provide further detail regarding the size and other relevant characteristics of the 29 maternity wards included in the study, including how the hospitals were chosen.*

R9: As requested further details are provided in the methods section, with details of individual units provided in a new supplementary Appendix (new Table 2) (lines 211 – 217 and new Appendix 2).

*C10. Methods and Results: The questionnaire administered at hospital discharge and 6 week follow up (mentioned at lines 161-163) should be included as a supporting document.  Please include a file containing the questionnaire, and provide a reference to it in the text.*

R10: As requested, the questionnaires have been added as supplementary tables 3 and 4.

*C11. Line 188: Please clarify the identity of the Chief Investigator.*

R11: As requested, the identity of the Chief Investigator has been clarified (line 292).

*C12. Line 188: Please clarify the identity, location, and the role of the “Trial Office”.*

R12: As requested, the identify, local and role of the “Trial Office” has been clarified (lines 288 – 291).

*C13. Line 225: Please remove the discussion of the cost analysis. Because the results of the trial were negative it is not necessary.*

R13: Further to our recent email correspondence with the handling Editor, and after discussion with our team, we believe that the cost analysis should be retained within our manuscript. However, we are happy to discuss further with the Journal if the Editors still request its removal.

As one of the interrelated domains of the primary outcome as stated in the study protocol, the authors firmly believe that the cost analysis should be retained in the manuscript. Despite negative clinical findings, circumstances can arise where cost-effectiveness findings still suggest value in utilising a technology. The inclusion of costs in the current manuscript refutes this possibility for GTN and helps to confirm the conclusions that GTN is neither clinically nor cost effective for medical treatment of retained placenta.

*C14. Line 239: Please remove the section “Role of the Funding Source” from the main text.  This is not needed as the information will be automatically extracted from the manuscript submission data.*

R14: As requested, we have removed this section from the main text.

*C15. Methods and Results: Please be consistent with numbers of decimal places when reporting p values.  In general, use two decimal places for p>.01 and three decimal places for p<.01 (e.g. Table 2 vs. Table 4).*

R15: We have amended as requested.

*C16. Discussion: Please qualify the first sentence of the discussion with the phrase “to our knowledge” or similar. (Line 339: “GOT-IT is the largest multi-centre, randomised, trial...”)*

R16: As requested, we have amended the first line of the discussion (line 494).

*C17. Discussion, Line 384: Please clarify what is meant by, and the role of the “lay advisors” mentioned.*

R17: As requested, we have clarified this statement in an expanded explanation in the discussion (lines 570 – 581).

*C18. Discussion: Please elaborate more on where and why the results may differ from previous research/studies of nitroglycerin treatment for retained placenta.*

R18: As discussed, this has been expanded in the discussion (lines 548 – 554 and lines 563 – 568)

*C19. Throughout the manuscript: Please use square brackets “[]” for in-text reference numbers, rather than parentheses.*

R19: As requested, the formatting of the references has been amended throughout.

*C20. For Supplementary Figures 1 and 2, please specify in the X axis label that ‘Sample Size’ denotes number of participants.*

We now provide a revised Figure 1. See also response to Reviewer 2, C5.

*C21. CONSORT checklist: Thank you for including the CONSORT checklist.  However, some of the page numbers are inconsistent, please double check and update throughout (e.g. Eligibility criteria for participants seem to be described on page 6, rather than page 7-10 as indicated).*

C22: As requested, the CONSORT checklist had been updated throughout.

Comments from the reviewers:

Reviewer #1:

*C1: This is, by far, the largest reported trial of glyceryl trinitrate (GTN) for treatment of retained placenta (RP) and the only trial of sub-lingual spray. The methodological quality of the trial is strong. I don't understand some of the statistical methods, but recognise that these will be reviewed by a statistical referee. The trial clearly needs to be published somewhere prominent, where it will be noticed.*

R1: We thank the reviewer for this supportive comment.

*C2: I do, however, think that the reporting of the trial could in some respects be improved. The relevant Cochrane review by Abdel-Aleem and colleagues (2015) - strangely uncited in the paper (it should be) - describes 3 randomised trials of GTN for RP (2 sub-lingual tablets, 1 intravenous); the combined incidence of manual removal of placenta (MROP) in the control groups was 80% and even though two of the trials had slightly more conservative time criteria for intervention (40 and 50 minutes) than GOT-IT, it is difficult to understand why the trial team planned on the basis of 50% MROP in the control group. This mis-match is only addressed in the paper in relation to the decision-making of the data monitoring committee in light of the unexpected incidence of the primary outcome. This should be discussed more fully.*

R2: The relevant Cochrane review is not included – we apologise for this omission. We have now included this.

At trial design, we carefully considered the existing evidence including the three trials included in the systematic review of Abdel-Aleem et al (1-3).

Bullarbo et al, 2005 (1) was a RCT conducted in a single hospital in Sweden. It included 24 women with placenta remaining undelivered 30 minutes after delivery, with women being randomised to receive either two 0.5 mg nitroglycerin (NTG) tablets taken sublingually), or placebo.

Bullarbo et al 2012 (2) was a RCT which included 111 women conducted in Sweden and compared the use 1 mg of sublingual NTG tablets, with placebo tablets administered 50 minutes after childbirth without delivery of the placenta.

Visalyaputra et al 2011 (3) was a RCT conducted in a tertiary hospital in Thailand and included 40 women with retained placenta and compared intravenous administration of NTG 200mcg with normal saline.

When both routes of administration (oral and iv) were considered, the three trials included 175 women, of whom 88 received placebo. When the two trials that used the oral/sublingual route of administration, were considered, there were 135 women, of whom 68 received placebo. As the reviewer indicates, two of the trials had a more conservative time for trial entry (40 and 50 minutes as opposed to 30 minutes) and none of the trials were conducted in the UK. Due to the quality and limited amount of data, it was clear that there was considerable uncertainty around the control rate, and whether the usual meta-analytic summary estimate would apply to our intended population in a UK setting.

From discussions with clinicians and women, we determined that an absolute benefit of 10% would be the minimum to make it worth implementing this intervention in practice. Our residual concern was then not to undertake an underpowered study. We therefore took a statistical approach to the control rate setting this at 50% because from a statistical perspective, this corresponds to the highest variability in a yes/no (binary) outcome, and hence generates the largest maximum sample size required to demonstrate an absolute 10% difference. That meant that we were safe in the knowledge that if the observed control rate was higher or lower, our study would be sufficiently powered to detect the original 10% absolute reduction and indeed potentially adequately powered to detect smaller absolute differences. Given the uncertainties in both the control rate and the minimum acceptable difference, this seemed a prudent strategy in designing this highly pragmatic study. As it turned out, we believe we were vindicated. We generated evidence that convincingly demonstrates that GTN is neither clinically nor cost effective, and we delivered this publicly funded study on time and within budget.

*C3: On a related topic, retained placentas are thought to either be detached but retained in the uterus (and potentially amenable to delivery after GTN relaxes uterine muscle) or non-detached (and much less likely to be aided by GTN). I don't know if there are good data on the incidence of each. My clinical impression is that the latter are much more common. I think these pathophysiological considerations need to be discussed in more depth to clarify the rationale for the trial, and to refine the discussion about sample size calculation.*

R3: We thank the reviewer for this comment.

The evidence for phenotyping retained placenta based on whether they are trapped, partially or completely adherent is largely based on clinical anecdote and at the time we conducted the study, there consensus about whether and how retained placenta should be classified and diagnosed. To our knowledge, this is still the case. Although ultrasound has been suggested for use in the area, there is no evidence that it is accurate for diagnosis or the results influence clinical management.

We have however refined our discussion to include the point raised by the reviewer (lines 556 – 568).

*C4: The rationale for the treatment regimen is not discussed in much detail other than saying that this was what the funder wanted for this commissioned research call. There are presumably data on bio-equivalence with other GTN regimens from eg internal medicine research. Why this treatment at this dosage? What evidence prior to trial commencement that this is efficacious? Current discussion could be expanded.*

R4: As requested, we have expanded this point in the discussion (page, line).

*C5: There are several references to the importance of RP in low and middle-income countries. The authors are correct to emphasise this. They might wish to expand a little on where they saw GTN having a possible role (should it have proven effective). Manual removal of placenta is one of the functions of the Basic Emergency Obstetric Care package, but access to health centres can often be problem. Did they see a potential role for medical intervention for RP in community care or in health centres or both?*

R5: If GTN had been efficacious and safe, we saw a potential utility for GTN in both community care and in health centres.

*C6: Surely the trial entry criterion 'delivery > 14 weeks' is a mistake (line 131)?*
R6: No, the inclusion criteria is correct. We were keen to allow women who were diagnosed with a retained placenta following a second trimester loss to take part in the trial. If GTN was effective in this group, it would avoid them having to go to theatre which would be particularly beneficial in this group of women who had had a fetal loss.

Jim Neilson


Reviewer #2:

This manuscript reports the results of a RCT comparing Glyceryl trinitrate to placebo for the treatment of retained placenta. The manuscript is well written and very easy to follow. I only have minor comments listed below:

*C1: In the method section, please indicate the nominal type-I error rate available at the end of the trial given the overall 5% level and the 5 interim reviews planned*

R1: As requested, this is now included in the methods, with the working level of significance being 0.048.

*C2: East 6.4.1 was used to analyse the primary clinical outcome. Please clarify the method (model, adjustment) used for the primary clinical outcome. In particular, please clarify whether the model adjust for the effect of centre?*

As requested, we have clarified the method used for analysis of the primary outcome

We intended to always adjust for centre as a random effect, and did so in all of the analyses except that of the primary outcome, which under the group sequential design was analysed in the EAST 6.4 software package, which did not support adjusting for centre. We are confident given the findings on the secondary outcomes and the adverse events that adjusting for the 29 centres with over 1000 participants will make negligible difference to either the point estimate or its 95% confidence interval, but we are happy to refit the model for the final analysis of the primary outcome in Stata if the Editors wish.

*C3: Please confirm that all patients with a CRF available post-discharge also had a primary outcome (MROP use) available*

R3: We can confirm that the completion for primary outcome was 100%

*C4: footnote in Table 2 states that "the analysis was adjusted for multiple looks at the data". Please clarify what this means. E.g. where the confidence intervals adjusted? the p-values? Is this true for all analyses presented in Table 2 or only the primary clinical outcome?*

Thank you for bringing this to our attention, and apologies for the confusion generated. The reviewer is correct, it is only the primary outcome that was under a group sequential design and hence needed to be adjusted for the multiple interim analyses. We have reworded the footnote to make it clear that this adjustment did not apply to the secondary outcomes.

We agree with the reviewer that for consistency we could or perhaps should have made a similar adjustment to the 95% confidence intervals by calculating them at the working significance level of the final analysis, given that the study continued to its maximum size without being stopped early for either overwhelming efficacy or futility – which would have equated to 95.2% confidence intervals. However, we took the view that adjusted confidence intervals are not widely understood, and in this particular case with very tight 95% confidence intervals this adjustment was negligible and so we did not apply it. We are happy to recalculate these 95% confidence intervals if the Editors think this will improve the statistical reporting.

*C5: The discussion states (lines 356-357) that "none of the efficacy and futility boundaries were crossed at any of the interim analysis" (a similar statement is made in lines 269-270); however, according to supplementary Figure 2, the futility boundaries were crossed at the 4th interim analysis. Please clarify/correct as well as explain more clearly this apparent inconsistency.*

We thank the reviewer for bringing to our attention the apparent inconsistency in Figure 2 (the plot graphing the scheduled interim analyses, including stopping early for efficacy or futility. This appeared to show that the data indicated that it might have been appropriate to stop for futility at the 4^th^ interim look. In fact, there was only one scheduled futility analysis, at the 3^th^ interim look; whereas at each of the 4 interim analyses before the final 5^th^ analysis there was the opportunity to stop for overwhelming evidence of effectiveness, there was only one opportunity to stop for futility. We should not have joined the dots from this futility analysis at the 3^rd^ look to the final analysis, thus creating the erroneous interpretation that the reviewer understandably formed. We have revised Figure 2 to make this clear.

Reviewer #3:

This is a very well-written and well-designed trial.  The clarity in writing and transparency in reporting is commendable.

I have only minor recommendations for this manuscript, which are as follows:

*C1: In the title or abstract, please include the term "nitroglycerin," to ensure broader understanding of glyceryl-trinitrate (GTN) as "nitroglycerin" is the more common term used in the U.S. and other countries.  Consider changing  the abbreviation "GTN" as this is commonly used to represent "gestational trophoblastic neoplasia"*

R1: As requested, we have amended throughout.

*C2: Lines 38-39 and 79-80: the authors discuss that the only alternative management for retained placenta is manual removal, and do not discuss curettage, a commonly performed procedure for retained placenta.  This, too, may not be available in lower resource settings, but deserves mention*

C2: As requested, we now include reference to this.

*C3: Lines 95-96: The authors correctly mention that glyceryl trinitrate may be effective for retained placenta in the presence of a contraction ring.  The authors appear to have used the medication as a solo measure.  A limitation of this study that is not mentioned, but that is more clinically relevant is how often nitroglycerin was needed to allow a contraction ring to relax sufficiently to allow passage of the providers hand to complete manual removal of the placenta, followed by uterotonics and therefore, reduce blood loss.  With this in mind, I recommend that the authors consider modifying their conclusion to state that glyceryl trinitrate is neither clinically effective nor cost effective when used alone, and either provide evidence that it is ineffective in this very clinically relevant subset or mention that it may still be considered in this setting, absent other recourse.*

C3: We used glyceryl trinitrate in combination with controlled cord traction, and not as a sole measure and have amended the conclusion to reflect this. In light of the safety signals (drop in blood pressure, rise in pulse and increased need for transfusion), and inability to diagnose a placenta which is trapped as opposed to being adherent, we have concerns about advocating the use of glyceryl trinitrate in any circumstances.

References

1.	Bullarbo M, Tjugum J, Ekerhovd E. Sublingual nitroglycerin for management of retained placenta. International journal of gynaecology and obstetrics: the official organ of the International Federation of Gynaecology and Obstetrics. 2005 Dec;91(3):228-32. PubMed PMID: 16226759. Epub 2005/10/18. eng.

2.	Bullarbo M, Bokstrom H, Lilja H, Almstrom E, Lassenius N, Hansson A, et al. Nitroglycerin for management of retained placenta: a multicenter study. Obstetrics and gynecology international. 2012;2012:321207. PubMed PMID: 22685465. Pubmed Central PMCID: 3364587.

3.	Visalyaputra S, Prechapanich J, Suwanvichai S, Yimyam S, Permpolprasert L, Suksopee P. Intravenous nitroglycerin for controlled cord traction in the management of retained placenta. International journal of gynaecology and obstetrics: the official organ of the International Federation of Gynaecology and Obstetrics. 2011 Feb;112(2):103-6. PubMed PMID: 21144515.
